# The Impacts of Dam Construction and Removal on the Genetics of Recovering Steelhead (*Oncorhynchus mykiss*) Populations across the Elwha River Watershed

**DOI:** 10.3390/genes12010089

**Published:** 2021-01-13

**Authors:** Alexandra K. Fraik, John R. McMillan, Martin Liermann, Todd Bennett, Michael L. McHenry, Garrett J. McKinney, Abigail H. Wells, Gary Winans, Joanna L. Kelley, George R. Pess, Krista M. Nichols

**Affiliations:** 1 School of Biological Sciences, Washington State University, Pullman, WA 99164, USA; joanna.l.kelley@wsu.edu; 2 Trout Unlimited, 1777 N. Kent Street, Suite 100, Arlington, VA 22209, USA; jmcmillan71@gmail.com; 3 Northwest Fisheries Science Center, National Marine Fisheries Service, National Oceanic and Atmospheric Administration, 2725 Montlake Boulevard East, Seattle, WA 98112, USA; martin.liermann@noaa.gov (M.L.); todd.bennett@noaa.gov (T.B.); garrett.mckinney@noaa.gov (G.J.M.); gary.winans@noaa.gov (G.W.); george.pess@noaa.gov (G.R.P.); 4 Lower Elwha Klallam Tribe Natural Resources, 760 Stratton Road, Port Angeles, WA 98363, USA; mike.mchenry@elwha.org; 5 Lynker Technologies, in Support of the Conservation Biology Division, Northwest Fisheries Science Center, National Marine Fisheries Service, National Oceanic and Atmospheric Administration, 2725 Montlake Boulevard East, Seattle, WA 98112, USA; abigail.wells@noaa.gov

**Keywords:** *Oncorhynchus mykiss*, population genetics, recolonization, anadromy, reduced representation sequencing, RAD sequencing, dam removal

## Abstract

Dam construction and longitudinal river habitat fragmentation disrupt important life histories and movement of aquatic species. This is especially true for *Oncorhynchus mykiss* that exhibits both migratory (steelhead) and non-migratory (resident rainbow) forms. While the negative effects of dams on salmonids have been extensively documented, few studies have had the opportunity to compare population genetic diversity and structure prior to and following dam removal. Here we examine the impacts of the removal of two dams on the Elwha River on the population genetics of *O. mykiss.* Genetic data were produced from >1200 samples collected prior to dam removal from both life history forms, and post-dam removal from steelhead. We identified three genetic clusters prior to dam removal primarily explained by isolation due to dams and natural barriers. Following dam removal, genetic structure decreased and admixture increased. Despite large *O. mykiss* population declines after dam construction, we did not detect shifts in population genetic diversity or allele frequencies of loci putatively involved in migratory phenotypic variation. Steelhead descendants from formerly below and above dammed populations recolonized the river rapidly after dam removal, suggesting that dam construction did not significantly reduce genetic diversity underlying *O. mykiss* life history strategies. These results have significant evolutionary implications for the conservation of migratory adaptive potential in *O. mykiss* populations above current anthropogenic barriers.

## 1. Introduction

Habitat fragmentation caused by anthropogenic disturbance is one of the largest contributors to loss of species and biodiversity globally [1,2]. Disruption of movement by human-made barriers has resulted in tremendous ecological and evolutionary consequences both in terrestrial and aquatic ecosystems [3]. The consequences may be particularly evident in dammed riverine ecosystems in which impassable barriers reduce habitat connectivity, thereby isolating populations, reducing gene flow and increasing genetic structure [4,5,6,7]. A lack of access to habitat for rearing, foraging, and reproduction can interrupt the expression of life histories, resulting in significant population declines [4,8,9]. In the long term, populations impacted by dams either adapt [10] or face extirpation [11,12]. Dams are particularly deleterious to migratory fishes as they prevent movement among different habitats necessary for various life history stages [13].

For salmonids, blockages of upstream habitat access for spawning adults and downstream access for juvenile out migration have been especially devastating consequences of dam construction [14]. Salmonids have high rates of natal homing, meaning they frequently return to their birth site to spawn [15]. Large geographic distributions across heterogeneous landscapes coupled with precise natal homing patterns have yielded a genetically and phenotypically diverse family of fish [16,17,18]. Fine-scale geographic structuring among populations and adaptation to local environmental conditions have also contributed to significant intraspecific diversity, producing a variety of life history forms [19,20,21]. These life history forms possess diverse migratory phenotypes that vary in their propensity for ocean migration, ocean return-timing, age-to-development, and migration patterns within freshwater [15]. These life history forms are also significantly stratified temporally and spatially, making species management and conservation challenging [18,22]. The study of partially anadromous species that exhibit multiple life history forms has thus been important for understanding the ecology and evolution of salmonids and their diverse migratory behaviors.

*Oncorhynchus mykiss* is a species that exhibits both an ocean-migrating (anadromous steelhead trout) and freshwater form (resident rainbow trout) [16]. While rainbow trout remain in freshwater throughout their lives, steelhead juveniles undergo significant physiological and morphological changes to prepare for marine conditions in a process called smoltification [23,24]. Mechanisms underlying these life history transitions are heritable [25,26], but also influenced by environmental conditions [27,28,29,30]. Anadromous steelhead and resident rainbow trout interbreed in sympatry [10,19,31,32], but experience significant genetic structure within [6,7,33] and between watersheds [34,35]. Genomic studies have identified targets of divergent selection among migratory life history strategies within a double inversion on chromosome 5 (Omy5) that contains genes functionally related to sexual maturation, development, and smoltification-related traits [36,37,38,39]. Deviations in these associations among higher-latitude populations, however, have suggested that non-parallel, population-specific mechanisms may be important in the expression of this migratory phenotype [39,40,41,42].

Steelhead also vary in temporal life history transitions including ocean migration patterns and return timing (run timing) [15]. Steelhead that return to spawning grounds in the summer and then mature sexually in freshwater over six to twelve months are known as summer-run steelhead, while those that enter freshwater close to or at maturity and spawn soon after are known as winter-run steelhead [15,43]. Previous work in *O. mykiss* found a major-effect locus for migration return timing in the *GREB1L* gene on chromosome 28 [44,45,46]. Variation in the *GREB1L* gene has also been identified as a candidate for adult run timing in Chinook salmon [45,47,48]; however, there is uncertainty regarding the extent to which variation in this gene explains run timing outside of coastal populations [49]. For example, in the Columbia River Basin, all inland steelhead are freshwater-maturing fish that would be predicted to possess the “summer-run” alleles observed in coastal populations [50]. However, both summer- and winter-run timing alleles have been detected in inland migrating populations and appear to be more strongly associated with arrival timing at spawning grounds rather than ocean migration timing [46]. Fragmentation of aquatic corridors and removal of natural barriers to migration have been implicated in the deviation of this genotypic–phenotypic association, indicating the importance of landscape connectivity for maintenance of run timing life histories [47,49,51,52]. Disruption to gene flow by barriers, therefore, could significantly impact the distribution of adaptive genetic variation underlying diverse migratory phenotypes.

Dams built without fish passage prevent ocean migration and restrict movement of aquatic organisms throughout the river. The construction of the impassable Elwha River and Glines Canyon dams, completed in 1913 and 1927, respectively, on the Olympic Peninsula north of Port Angeles, WA, present one such example. These dams fragmented the river into three sections: seven river kilometers (rkm) below the dams, 47 rkms in between the dams, and >70 rkms above both dams [53,54]. Ultimately, large population declines of anadromous fish populations, riverbank instability, coastal erosion, reduction in habitat quality, and public pressure culminated in the Elwha River Restoration Act in 1992 [53,55,56,57]. River restoration began with removal of the Elwha River and Glines Canyon Dams in 2012 and 2014, respectively, increasing access to over 120-river kilometers of historical spawning grounds to anadromous fishes [58,59,60]. Previous genetic work on *O. mykiss* in the Elwha River prior to dam removal identified genetic differentiation among populations separated by anadromous barriers, and natural and hatchery origin steelhead using thirteen microsatellites [6]. While this provided insight into how habitat fragmentation due to dams impacted *O. mykiss* population structure, the impacts of dam removal remain unexplored.

To fill this gap, we compared genetic and ecological life history diversity of *O. mykiss* before and after removal of dams on the Elwha River. In this study, we genotyped 71,320 SNPs from 567 samples of both steelhead and resident rainbow trout collected prior to dam removal and from 556 steelhead post-dam removal to investigate how steelhead are recolonizing the river. We hypothesized that if dams were the only barrier to gene flow between freshwater and anadromous life history forms of rainbow trout, then admixture should ensue following dam removal. If diverse migration and residency phenotypes existed across the Elwha River prior to dam construction, then we would expect diminished genetic and life history diversity in above-dam populations as a result of selection against the migratory phenotype. We thus expect recolonizing steelhead to be coming from populations previously restricted to below the dams that retained ocean access. Based on these initial hypotheses, we asked: (1) How does population genetic structure and diversity change following dam removal? (2) What was the source for adaptive alleles putatively involved in adult run timing (*GREB1L*) and anadromy (Omy5) post-dam removal? (3) What do these loci tell us about how steelhead are recolonizing the Elwha River watershed? The results from this study will provide crucial information for future decision making regarding the potential effects of dam removals for the conservation of diverse salmonid life histories.

## 2. Materials and Methods

### 2.1. Sample Collection

Samples were taken from 1,334 *Oncorhynchus mykiss* before (692 individuals from 2004 to 2011) and after (642 individuals from 2015 to 2019) the removal of the Elwha River and Glines Canyon Dams over a fifteen-year period (2004–2019). Both life history forms (anadromous and resident) of *O. mykiss* were sampled prior to dam removal (before 2011), across seven sites from above the dams, seven sites from in between the dams, one site above a natural waterfall, and one site from below the dams. We were primarily interested in characterizing and assessing whether returning steelhead post-dam removal were ancestors of pre-dam Elwha River collections of *O. mykiss*. Post-dam removal samples included only *O. mykiss* sampled 2015 and onwards when removal of the Glines Canyon dam was completed. Fin clips samples collected both prior to and post-dam removal were stored in 95% ethanol or on chromatographic blotting paper and quickly air dried.

**Figure 1:**
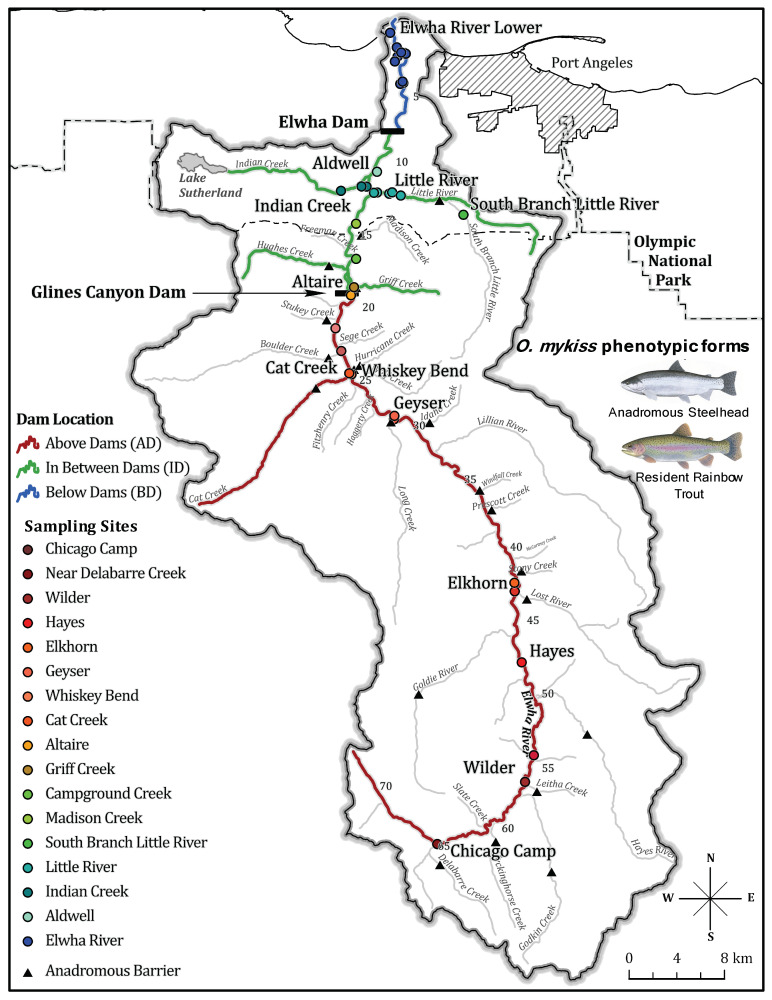
Map of all *Oncorhynchus mykiss* sampling sites across the Elwha River watershed on the Olympic Peninsula of Washington State. River segments are colored based off of relative dam location (population) and sampling locations are labelled and colored in a shade of the relative population color. Cartoons depict the two life history phenotypic forms of *Oncorhynchus mykiss* (art from graphics produced by Joseph Tomelleri) sampled in this study.

Prior to removal of the first dam, the lower Elwha River dam (2012), samples were obtained from sites on the main stem of the Elwha River as well as in tributaries. In future analyses, we divided sampling sites into three main sampling locations that were separated by impassable barriers: above both of the dams (AD), in between the dams (ID), and below the dams (BD) (Figure 1). The South Branch of the Little River (SBLR) is also separated from the Little River tributary of the Elwha River by an impassable, natural, three-meter waterfall and is also distinguished throughout as a separate sampling location. The four sampling locations (AD, ID, SBLR, and BD) are hereafter referred to as populations, while the 16 sampling sites remain known as sampling sites throughout the manuscript. Samples from this period were the same as those in Winans and colleagues’ microsatellite study on the Elwha River [6], with the exception of an additional collection from the Indian River.

Following the removal of the second dam, the upriver Glines Canyon Dam (2014), the lower (below both former dams) and middle portions (between both former dams) of the Elwha River were mainly sampled, with only three samples collected from the upper portion (formerly above the dams). Juvenile smolts were sampled in rotary screw traps from three sampling sites in 2016 and 2017: Little River (river kilometer (rkm) 0.2), Indian Creek (rkm 0.6), and on the main stem Elwha River (0.3–3.3 rkm) [61]. Adult steelhead were sampled during weekly or bi-weekly in-river tangle net sampling conducted at ten different sites in the lower, and to a lesser extent, upper and middle portions of the watershed [61]. Since smolts and steelhead were primarily sampled as they were migrating to or from the ocean, we excluded their sampling location as a factor in later analyses as it probably did not provide accurate data regarding their natal or spawning site. Therefore, post-dam removal samples were divided into life history cohorts by the estimated brood year for smolts, and the return year for adult steelhead. Both smolts and adult steelhead are identified as steelhead in later analyses.

Fin clips taken from *O. mykiss* fry and juveniles were acquired through electrofishing and smolt traps. These fish were designated as “unknown” life history phenotypes unless recaptured as adults. Juvenile *O. mykiss* captured in the rotary screw traps could be distinguished as smolts. Smolts were defined by length and coloration at the time of capture. During the early period of outmigration, for example, smolts still had faint parr markings. Fin edges during the early period were starting to darken, but were not fully black. In addition, the average smolt length was 165 mm, but could be as small as 87 mm and as large as 244 mm. During peak and late migration coloration was a fully silver bodied with a solid dark back and completely blackened fin edges and parr markings were very faint to completely gone.

Adult steelhead sampled post-dam removal included fish that were produced from naturally spawning adults as well as fish that originated from the Lower Elwha Klallam’s Tribe integrated hatchery program (Winans et al., 2017). This program used eyed eggs and trout fry from naturally spawning, native steelhead in the Lower portions of the Elwha River prior to dam removal (2005–2011) from below the lower Elwha River Dam (LEKT 2012). Samples from many of these naturally spawning parents were included in the below dam, prior to dam removal, collections included in this study, as well as previous genetic studies (Winans et al., 2017). In total, 209 steelhead sampled post-dam removal were identified as hatchery origin, 259 were of natural origin and 88 were of unknown origin. 

### 2.2. Library Preparation and DNA Sequencing

DNA from 1334 fin clip samples was isolated and extracted using the Qiagen DNeasy blood and tissue extraction kit (Qiagen, Valenica, CA, USA). dsDNA was quantified with Quant-iT picoGreen ds DNA Assay kit (Invitrogen, Watham, MA, USA) and UV spectrophotometry. All samples were normalized to 12.5 ng/μL, and 125 ng per sample was used for restriction site-associated DNA sequencing (RADseq) library preparation, using 96 samples per library. Briefly, RADseq was performed as described in [62] using the *SbfI* enzyme and sonication using the QSonica Q800R (QSonica LLC, Newtown, CT, USA) to selectively fragment the genome. RAD libraries were sequenced on an Illumina HiSeq 2500 or 3000/4000, pooling 2–3 libraries per lane, and indexed with NEBnext Multiplex i7 indices (New England Biolabs, Ipswich, MA, USA). Paired-end 100 base pair reads were obtained. After initial sequencing of pooled libraries, read counts were assessed, and in some cases, libraries were re-balanced and re-sequenced to acquire more data.

### 2.3. Read Alignment and Filtering

Data quality filtering and genotyping were conducted using the STACKS pipeline [63,64]. Process_radtags from Stacks v. 1.44 processed the forward and reverse reads for each sequencing lane to de-multiplex samples, quality filter the reads, and trim the reads to 85 bases. Since the *SbfI* site is found in both forward and reverse reads, process_radtags was run independently on these reads from each lane of sequencing, the *SbfI* site and barcode end identified. Data from the *SbfI* site were then combined into the R1 file for each sample, and the random fragmented sequence end concatenated into the R2 file for each sample. Following quality filtering, data were filtered to remove PCR clones using the clone_filter script from STACKS. Quality and clone filtered reads from each sample were aligned to the *Oncorhynchus mykiss* reference genome (NCBI: GCA_002163495.1 [39]) using default parameters in bwa [65]. Samtools was used to sort and index aligned reads from bwa, as well as remove unmapped and improper read pairs [66]. The resulting bam files were genotyped in STACKS (v. 2.2) using gstacks with default parameters. STACKS populations were used to collate genotypes across samples and populations, keeping only loci with a minimum of 65% of individuals genotyped in each population. In this case, we grouped all individuals into one population and therefore applied the filter to retain only loci genotyped in a minimum of 65% of the individuals. We used VCFtools [67] to filter the merged output file from the populations module in STACKS to remove non-biallelic sites, indels, sites with a minor allele-frequency <1%, sites with >10% missing data per SNP, and individuals with >20% missing data per sample. Due to presence of highly similar paralogs from the salmonid-specific genome duplication [68], we used HDplot to identify and remove possible paralogs using a combination of heterozygosity and read-ratio deviation [69]. Post-filtering, we retained 1125 individual *Oncorhynchus mykiss* (567 individuals from 15 sampling sites pre-dam removal and 556 individuals from five sampling sites post-dam removal) and 71,320 SNPs (Table 1). All further analyses, except for principal components analyses, were conducted on pre-dam and post-dam removal sample sets separately.

### 2.4. Principal Component Analysis (PCA)

We conducted a principal component analysis (PCA) using the R package Adegenet [70] to obtain an unsupervised visual assessment of genetic variance among individuals. We examined PCAs for all individuals sampled both pre- and post-dam removal using all SNPs. We also conducted PCA analyses for all individuals using SNPs restricted to chromosome 5 (Omy5) and chromosome 28 (Omy28) due to previous work detecting large-effect loci on these chromosomes [36,38,39,45,47].

### 2.5. Population Genetic Structure Analysis

We compared the results of two individual-based population assignment methods to determine whether the population structure that existed prior to dam removal remained after the dams were removed. Discriminant analysis of principal components (DAPC) in the R package Adgenet [71] uses K means clustering and model selection to estimate genetic clusters. We tested K (number of genetic clusters) values from 1 to 20 and selected the visual “elbow” or the lowest BIC value that described the genetic structure while minimizing the number of principal components retained. Optimal K values (number of genetic clusters) were those that yielded the greatest between group and least within group genotypic variation. We also ran fastSTRUCTURE [72] which uses a Bayesian framework to estimate the ancestry proportion for each individual for a given K value. We tested K values from 1 to 15 and selected the number of genetic clusters that maximized the marginal likelihood value. We ran each analysis on the pre- and post-dam removal data sets independently.

### 2.6. Population Genetics Analysis

We compared population genetic statistics from *O. mykiss* populations temporally to determine whether there were significant changes to genetic variation post-dam removal. All analyses were run separately across all pre-dam removal populations (AD, ID, SBLR, and BD) and post-dam removal life history cohorts separately (Adult Steelhead Pre-2015, Adult Steelhead 2015, Adult Steelhead 2016, Adult Steelhead 2017, Smolts 2016, Smolts 2017). We calculated population genetic differentiation (F_ST_; [73]), Tajima’s D [74] and nucleotide diversity (π; [75]) in VCFtools [67] to test for shifts in genetic variation that would be indicative of a shift in demographic structure. Tajima’s D and π were calculated by taking the average of 10 Kb (kilobase) windows to determine whether we detected a rapid signal of population contraction or expansion after dam removal.

Long-term conservation and management of *O. mykiss* requires information on how genetic variation and covariation among the sampled fish changed following dam removal. Therefore, we also calculated interpopulation relatedness (A_jk_), the inbreeding coefficient (F_IS_), and effective population size (N_e_) within populations prior to dam removal and within life history cohorts post-dam removal. Average relatedness was calculated in VCFtools (A_jk_ [67]) to determine whether individuals were more (A_jk_ range is between 0 and 1) or less (A_jk_ range is between 0 and −1) closely related to one another on average [32]. The inbreeding coefficient (F_IS_) was calculated using the R package Adegenet [70] to test for variation in observed heterozygosity. Finally, we tested for changes in N_e_ using N_e_Estimator v2 using the linkage disequilibrium parameterization [76]. Across population genetic statistics, with the exception of N_e_ which internally calculates confidence intervals, we performed non-parametric bootstrapping by resampling 10,000 samples from the original data set with replacement and generating 95% confidence intervals [77].

### 2.7. Overview of Genetic Stock Identification (GSI) Analyses

We used genetic stock identification (GSI) analysis to determine whether steelhead recolonizing the Elwha River following dam removal were produced from formerly above-dam populations. The R package RUBIAS [78] uses a conditional genetic stock identification model [79] to make Bayesian inferences about the genetic composition of individuals. We refer to the individuals used to derive reference populations as the “reference” sample set or individuals that are of known origin. We refer to the individuals we tested, of unknown origin, as the “mixed” sample set. RUBIAS allows for combination of populations of fish from various sampling sites (collections) into populations (reporting units) that are groups of fish that are genetically similar. The sampling sites were used as the collections for the reference samples in our study and were grouped into reporting units based on sampling location (AD, ID, SBLR, and BD). The mixed samples were coded as “NA” for reporting unit and divided into collections based on life history cohort.

In our GSI analysis, reference individuals were those sampled prior to dam removal with concordant assignment between population and DAPC group assignment; mixed individuals were all other samples. We additionally tested the inclusion of all individuals sampled prior to dam removal as the reference and all steelhead sampled post-dam removal were included in the mixed sample set. We thus divided individuals into two different reference and mixed sample sets hereafter referred to as sample set one and sample set two (additional details in the Appendix A). GSI analysis of the two sample sets were performed using SNPs that were selected to increase genetic differentiation between the reference reporting units (AD, ID, SBLR, and BD) prior to dam removal. We calculated Weir and Cockerham’s F_ST_ estimator [73] between each pair of reference reporting units) to identify the top 100 SNPs with the largest pairwise F_ST_. Since we used different individuals in each reference sampling set, we tested different SNPs with each sample set. We compared the accuracy of the two SNP panels by testing the SNPs used in the GSI analysis of the first sample set on the second sample set (additional details in Appendix A). Using pairwise reference reporting unit comparisons of F_ST_, we retained the unique, non-overlapping SNPs in each sample set, hereafter referred to as SNP set one (265 SNPs) and SNP set two (262 SNPs).

We compared the accuracy of our GSI SNP panels for each sample set by determining the probability of reference self-assignment using the “self-assign” function in RUBIAS. Additionally, we tested whether reference individuals in the second sample set had a higher probability of accurate self-assignment using SNP set one. We then conducted a GSI mixture analysis to determine the probability of the mixed samples from sample sets one and two assigning to reference collections. Additional details about determining self-assignment accuracy and assigning mixed samples to reference collections can be found in Appendix A. Hereafter, GSI results for the reference samples will refer to reporting unit as populations and collections sampling sites. Similarly, GSI results for the mixed samples will refer to reporting unit as inferred population and collection as life history cohort.

### 2.8. Evaluation of Candidate Loci for Involvement in Migration Phenotypes

#### 2.8.1. Candidate Genetic Variants for Migration and Run Timing

Finally, we investigated the distribution of adaptive genetic variation associated with *O. mykiss* migratory phenotypes among populations. We used twelve candidate loci located in a 55 megabase (Mb) double inversion on Omy5 with high genotyping rates across our samples and were previously shown to be associated with anadromy to look at genetic variation associated with the anadromous phenotype [38,39,80]. Due to the high levels of linkage disequilibrium observed with the Omy5 loci associated with the migratory phenotype, we summarized the twelve loci into a single Omy5 locus. We did this by summarizing the mean frequencies of the “A” type (ancestral/anadromous) alleles and “R” type (rearranged/resident) alleles [39] across all twelve loci. The frequencies “A” and “R” alleles were used to describe the Omy5 locus for each individual. We also investigated the frequencies of the “summer” and “winter” run timing alleles of a candidate SNP (Physical Position on Omy28 = 11,667,915) in the *GREB1L* gene on Omy28 to look at genotypic variation associated with migration run timing [44,47].

#### 2.8.2. Testing for Correlations of Putatively Adaptive Alleles and Migration Phenotypes

We used a two-sample *t*-test for equal means to first compare the average allele frequencies of our candidate loci between anadromy (Omy5 loci) and adult run timing (*GREB1L* locus) phenotypes (α = 0.01). Next, we performed generalized linear modelling in the R package lme4 [81] to test if Omy5 locus allele frequency (“A” VS “R”) explained life history phenotype (anadromous VS unknown). We used binomial models specifically, recoding steelhead with a value of “1” and unknown with a value of “0” for the life history phenotype. We also used a binomial model to test if *GREB1L* locus allele frequencies explained run timing phenotypes. Only steelhead were included in this analysis with summer-run steelhead recoded with a value of “1” and winter-run steelhead were recoded with a value of “0”. Contingency tables implement in the Genepop package in R were also conducted to test for allelic differentiation in the Omy5 and the *GREB1L* loci among life history phenotypes, populations, and time periods [82].

#### 2.8.3. Investigating the Geographic Structure of Adaptive Genetic Variants Involved in Migration

We next investigated whether the geographic structuring of Omy5 and *GREB1L* allele frequencies prior to dam removal were correlated with migratory phenotypes following dam removal. Since we did not necessarily have accurate information regarding natal/spawning population for most steelhead collected post-dam removal, we used population assignments from the GSI to identify inferred, “ancestral” population for steelhead collected post-dam removal. Steelhead were assigned to a single reference population if they had >90% shared genetic composition. All other steelhead were excluded from this analysis. We used these data to explicitly test two hypotheses regarding the source of putatively adaptive loci involved in migration on the Elwha River.

Our first hypothesis was that if below-dam populations retained ocean access, then we should detect higher frequencies of the “A” type Omy5 alleles. To test this, we performed a generalized linear model in the R package lme4 to determine whether Omy5 allele frequency variance explained the proportion of recolonizing steelhead from a given inferred population post-dam removal.

Our second hypothesis was that summer steelhead that migrate from the ocean earlier would move further upriver closer to the headwaters, therefore primarily coming from above-dam locations. To test this, we conducted an ANOVA in the R aov to determine whether populations location explained the proportion of summer alleles detected across populations.

## 3. Results

### 3.1. RAD-Sequence Filtering and Quality Control

After initial quality filtering of RADseq data, an average of ~1.5 million reads per individual were retained; removing PCR clones retained 960,812 reads for read mapping to the *O. mykiss* genome (Supplementary Table S1). We retained 1123 of the original 1334 individuals after filtering out individuals with high amounts of missing data, 567 of which were collected prior to dam removal and 556 steelhead following dam removal (Table 1). Reads mapped at an average rate of 97.3% per retained individual (Supplementary Table S1). After filtering, a total of 71,320 SNPs were retained for genomic analyses.

### 3.2. Principal Components Analysis (PCA)

There were some clustering patterns in the PCA related to population, but not to sampling site or migratory phenotype when including all loci sequenced among all individuals prior to dam removal (Supplementary Figure S1a) or post-dam removal (Figure S1b). The majority of the top 1% of SNPs that loaded on PC1 were on Omy5 and cumulatively explained the largest proportion of variance on this axis (Figure S1c). Similarly, the top 1% of SNPs that loaded on PC2 and PC4 were on Omy5, but the largest cumulative loadings were on Omy28. There was no distinct pattern on PC3 that could be associated with any single chromosome.

We observed three distinct clusters of SNPs on Omy5 loci that were primarily differentiated by PCs 1 and 3, but they did not appear to be associated with life history phenotype (Supplementary Figure S2). We similarly examined the patterns of clustering on Omy28 (Supplementary Figure S3) and did not observe any distinct clustering patterns.

### 3.3. Population Structural Analyses

**Figure 2:**
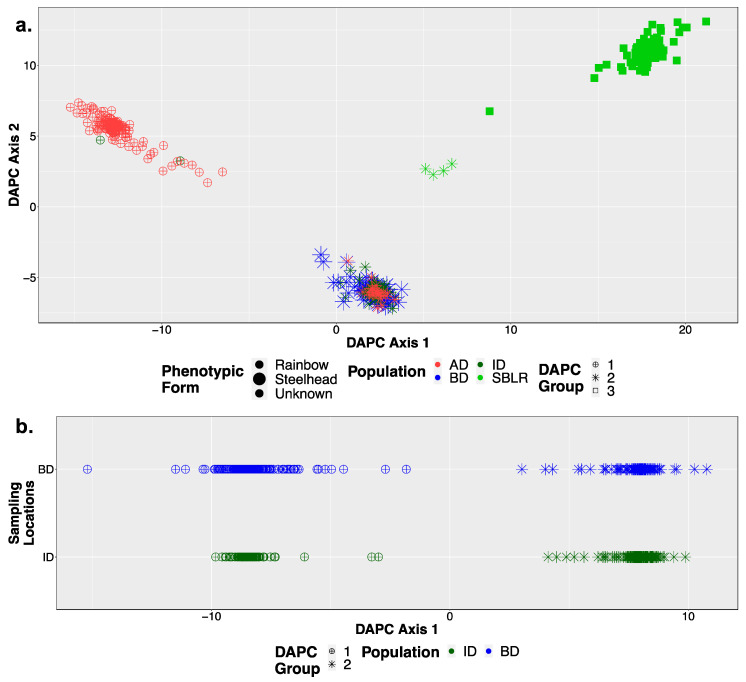
DAPC supported K = 3 genetic clusters prior to dam removal (**a**) and K = 2 genetic clusters following dam removal (**b**), note the points are split by sampling location for visualization. Shapes were indicative of population assignment by DAPC (DAPC Group) and color was based on population. Larger points in (**a**) were indicative of the steelhead life history phenotypic form while smaller points were indicative of the resident rainbow trout form or fish that could not be categorized in either form.

**Figure 3:**
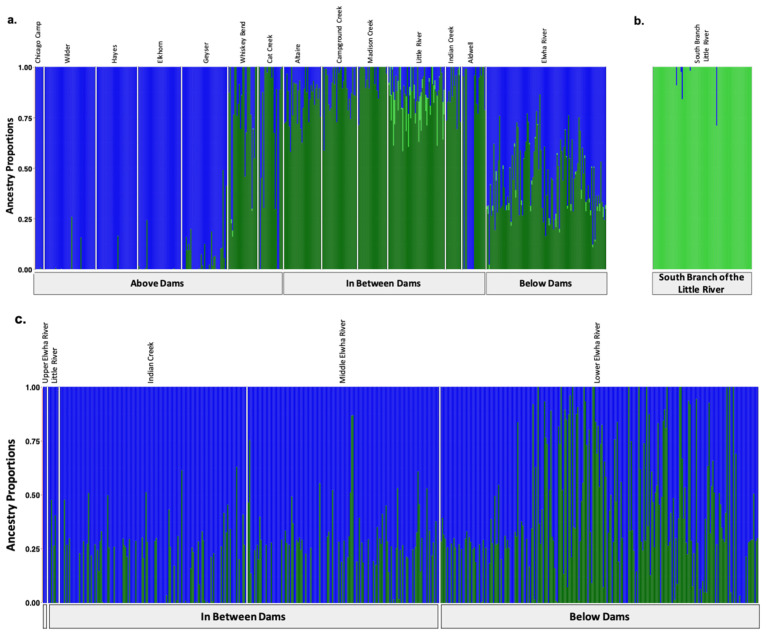
Population assignments computed by fastSTRUCTURE for samples collected (**a**,**b**) prior to dam removal supported three distinct genetic clusters (K = 3). (**a**) High levels of genetic structure were observed among populations split by man-made barriers such as dams (**b**) as well as natural barriers, such as the waterfalls that separate the South Branch of the Little River from the Little River. (**c**) Post-dam removal, we detected two genetic clusters with increasing levels of admixture. Each colored vertical bar represents a single individual sampled at one of the sampling sites labelled across the top x axis, organized from up-river to down-river and divided by anadromous barrier location, as labelled on the bottom x axis. The left-most samples in the plot, denoted by an unlabeled, empty gray box, include samples from above the dams, furthest up-river. Each color represents a distinct genetic cluster.

We identified three major genetic clusters prior to dam removal (Figure 2a) and two post-dam removal (Figure 2b) using DAPC. We observed three distinct genetic clusters along DAPC axis 1 separating the above the dams, the South Branch of the Little River, and the in-between-dam and below-dam populations (Figure 2a). Post-dam removal, however, we did not observe any genetic clustering by population along DAPC axis 1 (Figure 2b). FastSTRUCTURE similarly supported K = 3, with major differentiation occurring among populations geographically separated by anadromous barriers including the dams (Glines Canyon Dam) and waterfalls (South Branch Falls) prior to dam removal (Figure 3a). Post-dam removal, fastSTRUCTURE supported K = 2 with high levels of admixture among all individuals sampled (Figure 3b).

We also visualized K = 4 for DAPC (Supplementary Figure S4a) and fastSTRUCTURE (Supplementary Figure S5a,b) prior to dam removal, and K = 3 for both analyses post-dam removal as these were the next most well-supported models (Supplementary Figures S4b–d and S5c–e). We observed increased genetic structure among individuals sampled from the in-between-dam and below-dam populations in our fastSTRUCTURE plots for K = 4 (Supplementary Figure S5a,b) compared to K = 3 (Figure 3a,b). Post-dam removal, however, we did not see any clear increase in genetic structure among populations, life history cohorts or hatchery origin status between genetic structure plots for K = 3 (Supplementary Figures S4c,d and S5d) compared to K = 2 (Figure 2b and Figure 3c). We observed high levels of admixture and low genetic structure among individuals sampled post-dam removal for K = 2 and K = 3, suggesting high levels of gene flow among steelhead river wide post-dam removal.

### 3.4. Population Genetic Diversity on the Elwha River

#### 3.4.1. Natural Barriers and Dams Impacted Patterns of Genetic Diversity Pre-Dam Removal

**Figure 4:**
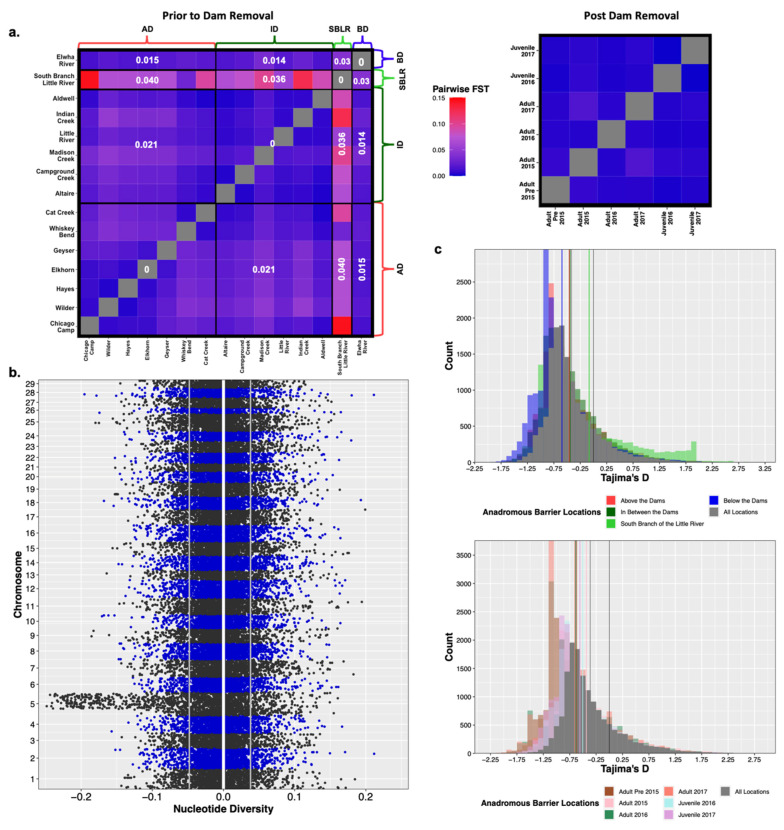
Prior to dam removal we saw genetic differentiation (F_ST_) among populations separated by anadromous barriers, but little temporal differentiation among life history cohorts sampled post-dam removal (**a**). We saw a significant increase in nucleotide diversity (π) at Omy5 post-dam removal, but not genome wide (**b**). The white vertical line is at zero, the two grey lines represent the upper and lower bounds of bootstrapped confidence intervals. There were statistically insignificant increases in estimates of Tajima’s D temporally and among life history cohorts sampled post-dam removal (**c**).

We observed genetic differentiation, as measured by F_ST_, among populations separated by dams and the South Branch of the Little River Falls (Figure 4a). However, we did not detect any significant variation in patterns of nucleotide diversity (π), inbreeding (F_IS_) or relatedness (A_jk_) that would suggest large differences in the amount of standing genetic variation among populations (Supplementary Table S2). We observed divergent patterns of population genetic diversity above the dams, in between the dams and below the dams, and the South Branch of the Little River. The South Branch of the Little River had the highest average estimates of inbreeding and relatedness, as well as the lowest estimates of effective populations size (N_e_), Tajima’s D and nucleotide diversity compared to the main stem of the Elwha River populations (Supplementary Table S2). The three main stem populations (AD, ID, and BD) had similar levels of standing genetic variation and had no significant variation among any population genetic statistic. All three populations also had similar, negative estimates of Tajima’s D that supported population expansion after a historic bottleneck. Although the below-dam population retained ocean access and the above- and in-between-dam populations did not, these populations had similar estimates of nucleotide diversity (π range = 0.052–0.061), relatedness (A_jk_ range = 0.0047–0.0090), and effective population size (N_e_ range = 120.9–150.7) (Supplementary Table S2).

#### 3.4.2. No Observed Shift in Population Genetic Diversity Post-Dam Removal

Of all genome-wide population genetic statistics measured, only effective population size (N_e_) exhibited statistically significant differences river wide temporally (Supplementary Table S2). However, neither the South Branch of the Little River nor resident rainbow trout were sampled post-dam removal, which may have downwardly biased estimates of effective population size. Additionally, small, uneven sample sizes of steelhead life history cohorts post-dam removal, may have also influenced our estimates of N_e_ as some had confidence intervals that had infinite upper limits (Supplementary Table S2). While we did not observe any genome-wide shifts in genetic diversity statistics, there were chromosomal differences in estimates of nucleotide diversity. Steelhead sampled post-dam removal had higher estimates of π on Omy5 compared to the genetic background (∆π Omy5 = −0.026 ± 0.07, ∆π genomic background = −0.0051 ± 0.043, Figure 4b). We also observed significant variation in estimates of N_e_ among life history cohorts post-dam removal (Supplementary Table S2). While Tajima’s D generally increased temporally post-dam removal among life history cohorts (Figure 4c), these differences were insignificant.

### 3.5. Genetic Stock Identification

#### 3.5.1. Assessment of the Accuracy of Our Reference Sampling Sites (Collections) and Populations (Reporting Units)

We retained 479 of the 567 (84.5%) individuals sampled prior to dam removal in reference sample set one that had concordant DAPC population assignment and sampling location (K = 4; Supplementary Figure S3). We used 265 unique SNPs in SNP set one to assign a total of 645 samples in mixed sample set one to reference reporting units (Appendix A). The mixed sample set included the 556 steelhead samples collected post-dam removal and the 88 individuals collected prior to dam removal, but not included in the reference set. On average, there was high probability of self-assignment of reference set individuals to their respective populations (average self-assignment probability = 0.86; Table 2), but lower power to self-assign individuals to their sampling site (average self-assignment probability = 0.55; Supplementary Table S3). Simulated individuals representing the reference set of individuals comprising the sampling sites and corresponding populations provided similar evidence for high correlation of true and simulated mixture proportions across populations (Supplementary Figure S6a,b). While the majority of simulated individuals had an average posterior probability of population assignment >90%, individuals simulated using mixing proportions from reference individuals sampled in Campground Creek, and the below the Elwha River dams deviated from this trend (Table 2). Individuals from these sampling sites had similarly low posterior probabilities of self-assignment to their respective sampling sites (Supplementary Table S3).

#### 3.5.2. Few Individuals Were Identified as of Potential Non-Elwha Origin

Six individuals out of the 645 tested mixture samples were not assigned to any single reference sampling site with significant confidence (Posterior probability of membership < 0.90). Three of these individuals were sampled prior to dam removal from in-between-dam populations and three were sampled post-dam removal (Supplementary Table S4). All analyses were restricted to the 639 individuals with high probabilities of membership to Elwha River sampling sites.

#### 3.5.3. Recolonizing Steelhead Cohorts Appeared to Be Derived from All across the River

**Figure 5:**
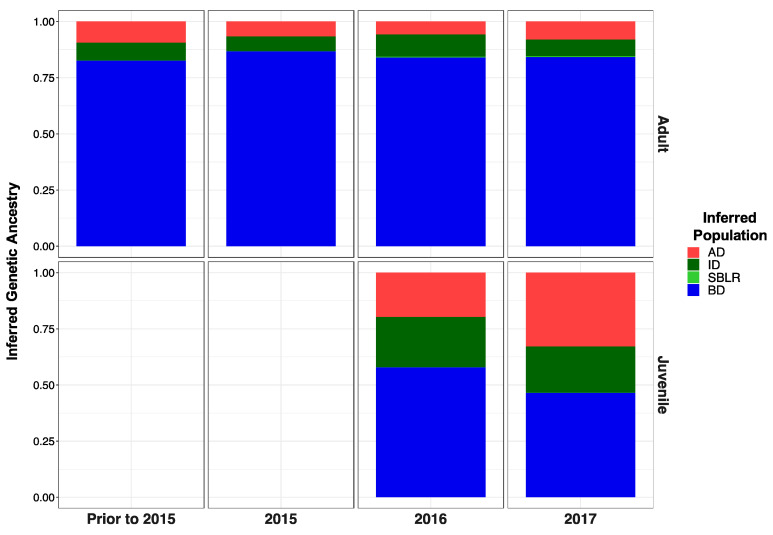
Inferred proportion of genetic ancestry from each of the four reference populations across recolonizing adult steelhead and juvenile smolt life history cohorts. Colors are representative of the inferred population. The bottom x axis divides life history cohorts temporally and the right y axis divides them by life history stage. We included the South Branch of the Little River (SBLR) as a control because we did not expect to see assignment to that population.

The majority of recolonizing steelhead across all life history cohorts appeared to be descendants of collections from below the dams (Figure 5). Over time, we saw increases in the proportion of ancestry from in-between-dam and above-dam collections; however, we had no individuals assign back to the South Branch of the Little River reporting unit genetic stock with any accuracy (Table 3). We saw substantial difference in the relative proportion of fish representing above-dam genetic ancestries between our life history cohorts, with adult returning steelhead having a larger proportion of below-dam genetic ancestries (Supplementary Figure S7) and juvenile smolt cohorts having larger proportions of above- and in-between-dam ancestry (Supplementary Figure S8).

Of the 567 individuals collected prior to dam removal, 88 individuals were not included in the reference sample set and tested in the mixture sample set. After removing the three individuals with low log-likelihood values (Supplementary Table S4), 86 samples were retained as mixture samples in our “pre-dam removal” life history cohort for GSI (Supplementary Table S5). A total of 33 of these individuals had concordant inferred population assignments to their actual population (Supplementary Figure S9). The other 52 individuals had discordant inferred population assignments from the actual population they were sampled from.

#### 3.5.4. Recolonizing Steelhead Cohorts Appeared to Be Derived from All across the River

**Figure A1:**
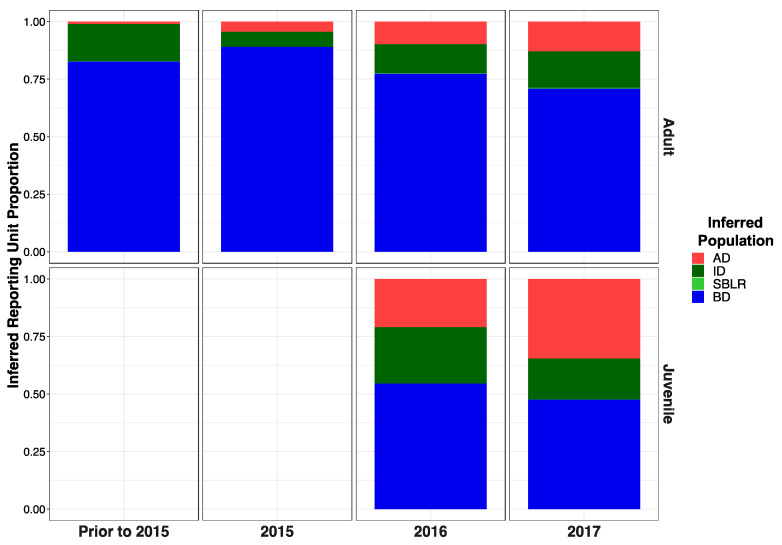
Inferred proportion of genetic ancestry from each of the four inferred populations across recolonizing adult and juvenile steelhead smolt life history cohorts from the GSI analysis (ii). Colors are representative of the inferred population. The bottom x axis divides life history cohorts temporally and the right y axis divides them by life history stage. Relative proportions of genetic ancestry from ID populations were greater in adult life history cohorts in GSI analysis (ii) compared to GSI analysis (i) and GSI analysis (iii).

**Figure A2:**
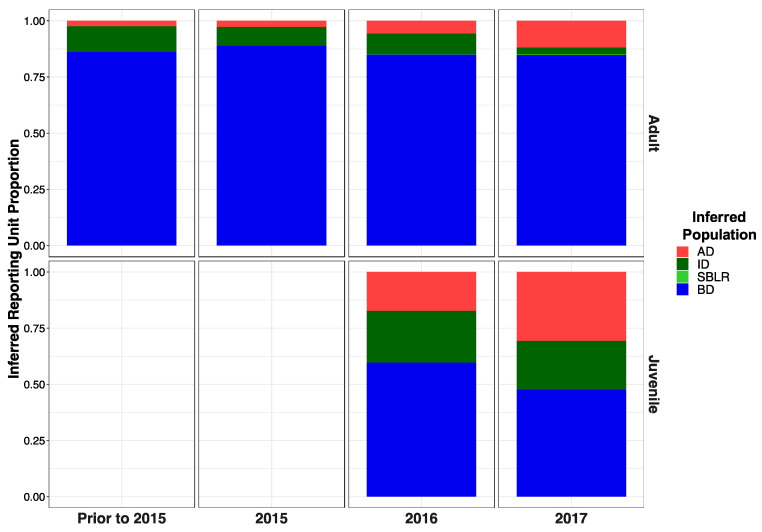
Inferred proportion of genetic ancestry from each of the four inferred populations across recolonizing adult and juvenile steelhead smolt life history cohorts from the GSI analysis (iii). Relative proportions of genetic ancestry from BD populations were greater in adult life history cohorts in GSI analysis (iii) compared to GSI analysis (i) GSI analysis (ii).

GSI results using sample set and SNP set 1 yielded the greatest power for self-assignment comparison to the other two GSI analyses conducted. We had the higher power to self-assign reference individuals back to their respective population and sampling site using SNP set 1 (Table A1) compared to SNP set 2 (Table A2). We identified one possible non-Elwha descendant fish using SNP set 1 (51686_15_87) and four possible non-Elwha descendant fish using SNP set 2 (51659_M_051216_smo_13, 51686_15_96, 51659_M_061516_smo_1, 51786_M_050217_9), none of which overlapped (Supplementary Table S4). Pre-dam removal samples could not be assessed as potentially non-Elwha in these GSI analyses, however, since all 567 individuals were included in the reference sample set. Across all three GSI analyses, however, mixed sample life history cohorts contained similar relative proportions of genetic ancestry from AD, ID, and BD populations (Figure A1 and Figure A2).

### 3.6. Allelic Variation in Candidate Loci for Migratory Life History Adaptations

#### 3.6.1. Temporal and Geographic Variation in the Distribution of Adaptive Migratory Potential

**Figure 6:**
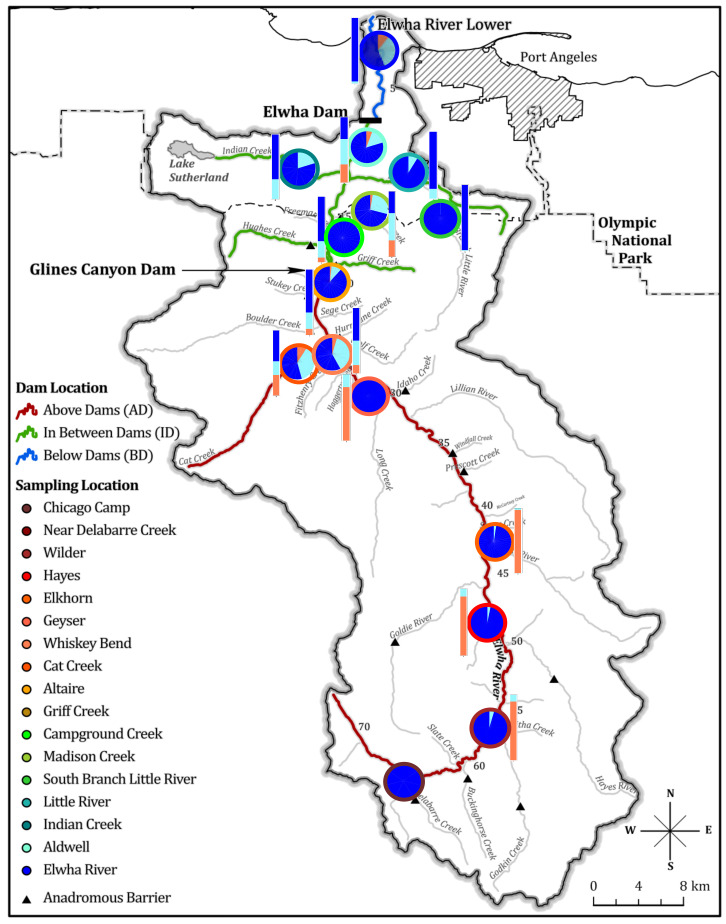
There was significant geographic variation in genotype frequencies for the Omy5 consensus locus (pie charts) and a candidate *GREB1L* variant (bar chart). The dark blue represents the resident homozygous genotype for the Omy5 consensus locus and the winter homozygous genotype for the *GREB1L* variant. The coral color represent the anadromous homozygous genotype for the Omy5 consensus locus and summer homozygous genotype for the *GREB1L* variant. The light blue represent the heterozygous genotypes. Chicago Camp did not have a sufficient number of individuals genotyped for the *GREB1L* variant to include a bar chart.

We observed substantial geographic variation in the Omy5 and *GREB1L* loci among the genotypes of populations sampled prior to dam removal (Figure 6). There was little variation in the Omy5 genotype in the majority of the above-dam sampling sites with fixation of the “RR” genotype in the Geyser and Chicago Camp (Figure 6). The population below the dam had the highest frequency of the anadromous “AA” Omy5 genotype but had very little variation in the frequency of the “summer” allele in the *GREB1L* variant. We observed global allelic differentiation (*p* < 0.001) among life history forms and populations prior to dam removal, and temporally at 11 out of the 12 loci (*p* > 0.01, Omy5_55408270) and at the *GREB1L* variant. Similarly, we observed global allelic differentiation (*p* < 0.001) post-dam removal among life history cohorts at 11 out of the 12 loci (*p* > 0.01, Omy5_55408270), and at the *GREB1L* variant. We also observed allelic differentiation (*p* < 0.001) among phenotypic forms at the *GREB1L* variant.

**Figure 7:**
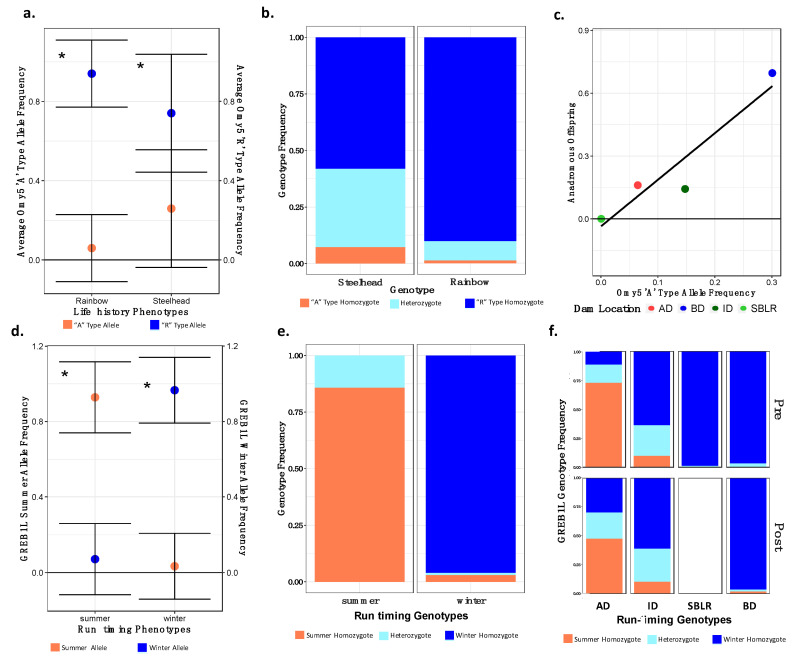
There were significant differences in the allelic and genotypic frequencies of the Omy5 (**a**–**c**) and the *GREB1L* variants (**d**–**f**) among populations and life histories. There were significant differences in the mean allele frequencies of the Omy5 locus allele frequencies between steelhead and unknown forms (**a**) and the frequency of the Omy5 “A” type allele frequency significantly explained life history phenotype (**b**). We also observed a strong correlation between the number of anadromous individuals from each population and the frequency of the “A” allele (**c**). The mean frequency of the *GREB1L* summer allele also varied significantly between summer- and winter-run steelhead (**d**), with the frequency of the homozygous summer genotype significantly explaining run timing (**e**), and inferred, ancestral population explaining a significant proportion of the variation in genotype both prior to and post-dam removal (**f**). Asterisks (**a**,**c**) indicate statistical significance (α = 0.01) (*).

We identified differences in the mean “A” (two sample *t*-test: −14.16, df = 1084.5, *p* < 0.001) and “R” type (two sample *t*-test: *t* =14.16, df =1084.5, *p* < 0.001) Omy5 allele frequencies between steelhead and unknown *O. mykiss* phenotypes (Figure 7a). Similarly, we found that the steelhead phenotypic form was explained by the “A” allele frequency (binomial model: β = 3.601, z = 11.426, *p* < 0.001) and was negatively correlated with the “R” Omy5 allele frequency (binomial model: β = −0.165, z = −2.253, *p* = 0.024) (Figure 7b). However, we identified a higher frequency of “RR” genotypes on average in steelhead than “AA” genotypes both pre- (AA = 0.11, RR = 0.56) and post-dam removal (AA = 0.07, RR = 0.58). There was also a weak, non-significant, relationship between the interaction of inferred population and the “A” allele frequency and the proportion of anadromous individuals produced (generalized linear model: β = 2.286, *t* = 4.047, *p* = 0.056). These results provided support for our hypothesis that the below-dam population had the highest frequency of “A” alleles and produced the largest proportion of anadromous individuals (Figure 7c). The South Branch of the Little River population was fixed for the “R” type allele as we might expect.

#### 3.6.2. Variation in the *GREB1L* Candidate for the Run Timing Phenotype

We found mixed statistical results to support our hypothesis that the above-dam population would harbor higher proportions of the *GREB1L* summer-run timing allele. We found differences in the mean associations of the *GREB1L* summer (two sample *t*-test: −14.16, df = 1084.5, *p* < 0.001) and winter (two sample *t*-test: *t* =14.16, df = 1084.5, *p* < 0.001) alleles between run timing phenotypes in steelhead (Figure 7d). Although we had a small sample size of known summer steelhead post-dam removal (n = 7), we found that the *GREB1L* genotype explained a significant proportion of the variance in the run timing phenotype (binomial model: β = 6.281, z = 3.936, *p* < 0.001) (Figure 7e). Furthermore, results from our ANOVA supported that the frequency of *GREB1L* summer alleles varied among populations before dam removal (ANOVA: df = 3, F = 418.3, *p* < 0.001). The above-dam population, in particular, had the highest frequencies of the summer-run alleles both prior to and post-dam removal (Figure 7f).

## 4. Discussion

In this study, we observed significant shifts in genetic structure in *Oncorhynchus mykiss* populations on the Elwha River within two generations following dam removal. Prior to dam removal, we observed genetic structure consistent with the findings from Winans and colleagues [6] illustrating broadly that the microsatellite and RADseq data sets similarly identified genetic structure associated with anadromous barriers including both dams and waterfalls. After dam removal, we found that genetic structure diminished after connectivity was restored. There were no significant temporal shifts in genetic diversity detected following dam removal. However, we found evidence supporting the initiation of population expansion in main stem populations after a historical bottleneck. Adult steelhead cohorts returning to the Elwha River to spawn following dam removal were primarily descendants of below-dam populations. However, out-migrating smolt cohorts had increased proportions of genetic ancestry from the above- and in-between-dam populations. The Omy5 and the *GREB1L* loci were associated with anadromy and adult run timing, respectively. However, the strengths of those genotypic–phenotypic associations were variable and will require further investigation over time as summer-run steelhead increasingly return to the upper watershed. Although dam construction led to declines in anadromous fish abundance, our findings suggest that the dam construction did not diminish genetic variation underlying the steelhead life history form on the Elwha River.

### 4.1. Natural Barriers Had a Greater Effect on Patterns of Genetic Variation Than Dams

Like previous studies, we found less genetic structure within river reaches separated by anadromous barriers compared to among them [33,35,83]. Population structure results supported three to four genetic clusters prior to dam removal separated by permanent anadromous barriers (Figure 2a, Supplementary Figure S4a), similar to previous microsatellite work in the Elwha [6]. Two of these barriers, the lower Elwha River Dam and upper Glines Canyon Dam on the main stem of the Elwha River, were impassable for upriver migration of returning steelhead and restricted downriver out migration of smolts [57,58,59]. Population genetic diversity results for the dam-fragmented population were mixed, showing genetic differentiation (Figure 4a), but similar levels of genetic diversity among populations before and after dam removal (Supplementary Table S2). Additionally, varying degrees of genetic admixture were observed within some reaches (Figure 3a). For example, two above-dam sampling sites, Cat Creek and Whiskey Bend, were genetically admixed with in-between-dam genetic ancestries (Figure 3a, Supplementary Figure S5a,b). These findings were concordant with Winans and colleagues’ findings, most likely reflecting a historic population break generated by a flow velocity barrier Rica Canyon [6,84].

A natural, three-meter waterfall separates the South Branch of the Little River population from other populations. Although steelhead have been documented to jump to heights of four to five meters, they require jump pools that are 1.25 times deeper than the jump height in order to generate the force necessary to propel themselves over these barriers [85]. The “undersized” jump pool at the base of the falls is so shallow that it has most likely prevented historic and contemporary recolonization of this tributary, thereby isolating the fish from other main stem populations (personal communication John McMillan). In addition to increased genetic structure, we also observed highly divergent patterns of population diversity and demography in the South Branch of the Little River compared to main stem populations. Previous work on *O. mykiss* in the Russian River basin in California similarly detected increased genetic differentiation among fish separated by natural barriers rather than man-made ones [35]. Although the dams were present for over 100 years (20–25 generations), our results suggest that populations divided by dams were not as diverged as those separated by natural barriers. Historic isolation of the South Branch of the Little River population from other main stem population by natural barriers most likely downwardly biased estimates of N_e_ and Tajima’s D based on life history forms and cohorts (Supplementary Table S2).

### 4.2. Population Genetic Structure Eroded Rapidly Following Removal of the Dams

Post-dam removal, genetic structure decreased, with no clear temporal, geographic or life history factors appearing to describe patterns of admixture (Figure 3c, Supplementary Figure S4c). Increased frequencies of below- and in-between-dam genetic ancestries post-dam removal supported GSI results that recolonizing steelhead are descendants of *O. mykiss* populations from Elwha populations (Figure 3c, Supplementary Figure S5d). Slight increases in Tajima’s D paired with observations of increasing steelhead populations on the Elwha River would suggest rapid population rebound following dam removal [61]. These findings support our initial hypothesis that dams were major barriers to gene flow. However, since only steelhead were sampled post-dam removal, population genetic structure among life history forms may not have been captured in our analyses [31,86]. Winans and colleagues, for instance, identified significant genetic differentiation between upriver and middle river *O. mykiss* to below-dam steelhead on the Elwha River populations prior to dam removal [6]. However, we did not detect significant genetic differentiation among steelhead and non-steelhead life history forms prior to dam removal, similar to observations of *O. mykiss* in other dammed ecosystems [10,19,34,87]. Therefore, we expect our observed patterns of admixture to reflect genetic exchange among recolonizing steelhead with resident rainbow trout following dam removal.

### 4.3. Recolonizing Steelhead Appear to Primarily Be Derived from the Elwha River Watershed

GSI analyses supported our hypothesis that adult steelhead recolonizing the Elwha River for the few years after dams were removed (2015–2017) were primarily produced from the below the dams (Table 3). We observed minimal contributions of genetic ancestry from the South Branch of the Little River reference population to the returning steelhead, concordant with our expectations. The “A” type or anadromous alleles for the Omy5 loci were observed in all three main stem populations prior to dam removal (Figure 6) in contrast to previous findings that observed latitudinal restrictions to the association of Omy5 alleles with migration [39].

Although adult steelhead cohorts lacked variation in GSI assignment of inferred population, out-migrating smolt cohorts (2016–2017) had high proportions of above- and in-between-dam genetic ancestries (Supplementary Figure S8). With the removal of the lower dam completed in 2012, the upper dam in late 2014, and the boulder barrier near the former Glines Canyon Dam removed in 2015, the earliest we may expect above-dam populations to receive homing steelhead to spawn would have been 2016 [88]. Recolonizing steelhead with upriver genetic ancestry in earlier cohorts may have been produced by individuals that passed as juveniles over the dams during high water flow (Personal Communication Mike McHenry; [89,90]). Resident *O. mykiss* upstream of Glines Canyon Dam had historically produced migrants that were seawater tolerant and apparently capable of an anadromous life history as late as the early 1990′s [91]. While some outmigrating steelhead may have passed over the dams prior to removal, these patterns more likely reflect the rapid rebound in smolt productivity of these formerly dammed populations. This has important management applications as it may indicate increased expansion of recolonizing steelhead into the upper portions of the Elwha River watershed.

Across all of our GSI analyses, we did not detect more than five fish that appeared to be of non-Elwha origin (Supplementary Table S4). As we hypothesized initially, some recolonizing steelhead may have strayed from nearby watersheds. Although steelhead and other anadromous salmonids are known to return to their natal sites at high rates [92], they have been observed to exhibit density-dependent straying [93]. Another possibility is that there may have been undocumented stocking or translocations of fish from nearby watersheds into the upper or middle portions of the Elwha River. Stocking of *O. mykiss* in high-elevation lakes above-dam and in-between-dam high-elevation lakes was recorded on the Elwha River prior to dam removal until 2001 [59]. Goldendale Hatchery trout sourced from California were also supplemented to an in-between-dam location, Lake Sutherland, from 1995 until 2012 [6]. Movement of hatchery stock from this lake into Indian Creek and other in-between-dam tributaries was prevented by a rotating screen. Additionally, while the Chambers Creek hatchery program ceased in 2011, a native broodstock steelhead hatchery program began in 2005 (releases began in 2006) on the Lower Elwha and continues to release fish. However, both previous work and our analyses have not identified significant impacts of the Chambers Creek program nor the current hatchery program on the genetics of the native steelhead population (Winans et al. 2017; unpublished data). Although we did not include samples from any locations that were known to have been supplemented recently, we cannot necessarily rule out the possibility that fish were translocated by humans, animals or highwater flows. Low probability of assignment of these steelhead to inferred populations may also be the result of interspecific hybridization, as has been previously observed in steelhead and sympatric cutthroat trout (*Oncorhynchus clarkii*) subspecies [94].

### 4.4. Patterns of Genetic Diversity Did Not Change Following Dam Construction or Removal

In spite of extensive population declines in *O. mykiss* and other Pacific salmonids in the Elwha River following dam construction, we did not detect significant spatial or temporal changes to population genetic variation suggestive of significant dam-mediated evolution of *O. mykiss* populations on the Elwha River. Indeed, recolonizing steelhead appeared to primarily be derived from Elwha populations harboring similar levels of genetic diversity and adaptive genetic variation. One reason for this may have been related to the time frame. Although the dams were in place for over 100 years, selection may not have had the time to remove the genetic variation underlying the migratory phenotypes from above-dam populations [95,96].

Another possibility is that the candidate loci identified in previous studies for their involvement in life history [38,39,80] were not associated with migration in this watershed. Although we detected significant differences in the Omy5 anadromous allele frequency between life history forms, this association was weak, suggesting a less important role of these alleles in the expression of the anadromous phenotype in Elwha River steelhead (Figure 7a). The strength of this association may have been impacted to some extent by our lack of phenotypic data on juvenile trout that were not resampled as adults. However, this is probably unlikely as we observed low frequencies of the anadromous allele in Omy5 loci in known steelhead (Figure 7b). Weinstein and colleagues also found no association between Omy5 haplotype and life history form in *O. mykiss* populations in southeastern Alaska, suggesting a latitudinal limit to this genotypic–phenotypic association with anadromy [42].

These loci may also be pleiotropic and maintained by positive selection. Although we had a small sample size of recolonizing summer-run steelhead, we identified significant variation in the frequency of the *GREB1L* alleles with run timing phenotypic forms (Figure 7d,e). We also observed higher frequencies of the *GREB1L* summer allele among above-dam populations prior to and post-dam removal closer to the snow melt-fed headwaters (Figure 7f), where historically summer steelhead have preferentially spawned [88]. Local selection in these colder, upriver environments, therefore, may favor the maintenance of summer-run steelhead and the standing genetic variation associated with these phenotypic forms.

The short time scale between dam removal and sampling may also have influenced our power to detect changes in genetic diversity and variation. Taking into account that the first dam removal began in 2011, one to two generations at most have passed since our last sample was collected for the in-between-dam population. The three-year delay until the removal of the upriver dam coupled with the significant variation in *O. mykiss* age-to-maturity [49,97], however, suggests that this may be a generous estimate. Significant, sustained, shifts in effective population size for several generations is required in order to observe significant shifts in genetic diversity [98]. Therefore, one possibility is that we could be observing the initial stages of evolutionary changes in life history variation in *O. mykiss* as a result of long-term isolation. Since only steelhead life history forms were sampled post-dam removal, observed admixture may only be occurring between historic steelhead due to reproductive barriers. After over 100 years of isolation, adaptation of above- and in-between-dam populations to a resident lifestyle may result in genetic incompatibilities or deviations in mating behavior or timing that limit or inhibit admixture. Assortative mating would increase genetic structure and differentiation and slow down steelhead recovery on the Elwha River. While we think this is unlikely due to a lack of detected differences in genetic diversity among steelhead and non-steelhead life history forms prior to dam removal, this should be examined in future studies.

## 5. Conclusions

As awareness of the deleterious effects of dams on migratory species have increased, so have the efforts to facilitate reconnection (e.g., fish ladders, fish reintroduction) or remove these obstructions altogether (e.g., dam removal). Dam removal case studies offer the unique opportunity to evaluate the effects of dam construction on species’ population genetics. Previous studies have found that populations of anadromous fish above human-made barriers, such as dams, do not necessarily have reduced genetic diversity. While these populations often experience significant population declines, maintenance of standing levels of genetic variation at loci putatively involved in anadromous phenotypes leaves the potential for rapid reestablishment of formerly dammed populations with anadromous life history forms of salmonid fishes, such as steelhead. This study characterizes the impacts of dam removal on the genetic variation of *O. mykiss* on the Elwha River. We show that recolonizing steelhead were primarily derived from Elwha River populations, allowing for rapid recovery of summer-run steelhead post-dam removal. Population declines followed dam construction and genetic structure increased among populations separated by dams. There were no significant shifts in genetic diversity among the dam-fragmented population, however, suggesting that genetic structure was most likely generated as a result of stochastic forces. Decreased genetic structure and admixture occurred rapidly following dam removal, suggesting no behavioral or reproductive barriers evolved during isolation. Future studies should estimate the strength of selection of dams on genetic variation underlying life history phenotypic variation to provide guidance for future dam removal and conservation projects.

## Figures and Tables

**Table 1:** All sequenced samples arranged by population (AD, ID, SBLR, and BD) and sampling site, ordered from upstream (top) to downstream (bottom) divided by relative anadromous barrier location.

Relative Dam Location Sampling Site	Prior to Dam Removal	Post Dam Removal
**Above the Dams (AD)**	**208**	**3**
Cat Creek	21	
Chicago Camp	7	
Elkhorn	37	
Elwha River		3
Geyser	39	
Hayes	35	
Whiskey Bend	25	
Wilder	44	
**In between the Dams (ID)**	**169**	**304**
Aldwell	20	
Altaire	32	
Campground Creek	30	
Elwha River		150
Indian Creek	13	146
Little River	49	8
Madison Creek	25	
**South Branch of the Little River (SBLR)**	**86**	**0**
**Below the Dams (BD)**	**104**	**249**
**Total**	**567**	**556**

**Table 2:** Simulations performed on all 479 individuals included in the reference sampling sites reflected high accuracy of assignment on average across individuals from each sampling site back to their respective population (AD = above the dams, ID = in between the dams, SBLR = South Branch of the Little River, and BD = below the dams). The posterior likelihoods for population assignment displayed below reflect the mean of the scaled likelihoods of assignment of individuals to an inferred population based on sampling site. The scaled likelihood of individual assignment is calculated by taking the sum of the likelihoods of assigning a fish back to their inferred sampling site. Bolded values indicate the highest mean posterior likelihood for inferred population assignment.

Sampling Site	Population	Inferred Population			
		AD	ID	SBLR	BD
Chicago Camp	AD	**1.000**	0.000	0.000	0.000
Wilder	AD	**1.000**	0.000	0.000	0.000
Hayes	AD	**1.000**	0.000	0.000	0.000
Elkhorn	AD	**1.000**	0.000	0.000	0.000
Geyser	AD	**1.000**	0.000	0.000	0.000
Cat Creek	AD	**1.000**	0.000	0.000	0.000
Altaire	ID	0.004	**0.996**	0.000	0.000
Campground Creek	ID	0.105	**0.895**	0.000	0.000
Madison Creek	ID	0.000	**1.000**	0.000	0.000
Little River	ID	0.000	**1.000**	0.000	0.000
Indian Creek	ID	0.000	**1.000**	0.000	0.000
Aldwell	ID	0.000	**1.000**	0.000	0.000
SBLR	SBLR	0.012	0.000	**0.976**	0.012
Elwha River	BD	0.148	0.020	0.000	**0.833**

**Table 3:** The mean probability of assignment to each population represented in the steelhead life history cohorts sampled post-dam removal varied temporally. The below-dam population represented the largest proportion of genetic ancestry in the first steelhead life history cohorts recolonizing the Elwha River following dam removal with increased representation from above- and in-between-dam populations.

Mixture Collection	Mean Posterior Probability				Mean Posterior Probability Credible Interval			
	AD	ID	SBLR	BD	AD	ID	SBLR	BD
**Adults Pre 2015**	0.094	0.0789	0.001	0.825	0.035–0.183	0.024–0.165	0–0.012	0.716–0.913
**Adults 2015**	0.067	0.065	0.000	0.868	0.034–0.109	0.032–0.105	0–0.005	0.813–0.916
**Adults 2016**	0.057	0.100	0.003	0.840	0.004–0.175	0.016–0.239	0–0.035	0.673–0.956
**Adults 2017**	0.080	0.074	0.003	0.842	0.006–0.246	0.005–0.223	0–0.035	0.644–0.969
**Juveniles 2016**	0.198	0.223	0.000	0.578	0.141–0.257	0.164–0.288	0–0.004	0.502–0.654
**Juveniles 2017**	0.330	0.206	0.001	0.464	0.243–0.414	0.138–0.282	0–0.006	0.374–0.562

## Data Availability

The sequence data has been deposited at NCBI under BioProject PRJNA690579. Code is available at github.com/jokelley/population-genetics-elwha.

## Supplementary Materials

The following are available online at https://www.mdpi.com/2073-4425/12/1/89/s1, Figure S1: Principal Components Analysis Including All Loci; Figure S2: Principal Components Analysis Including Only Omy5; Figure S3: Principal Components Analysis Including Omy28; Figure S4: Discriminant Analysis of Principal Components of Pre- and Post-dam Removal Samples; Figure S5: Fast Structure Analysis of Pre- and Post-dam Removal Samples; Figure S6: Genetic Stock Identification Simulation Results; Figure S7: Genetic Stock Identification Assignments of Adult Cohorts; Figure S8: Genetic Stock Identification Assignments of Smolt Cohorts; Figure S9: Inferred Proportions of Genetic Ancestry across life history cohorts; Fish; Table S1: Alignment Statistics Summary; Table S2: Mean Population Genetic Statistics; Table S3: Genetic Stock Identification Self-Assignment Simulation Results; Table S4: Unassigned Individuals from Genetic Stock Identification Analysis; Table S5: Probability of Membership Assignment for Discordant Individuals.

## Appendix A

### Appendix A.1. Reference and Mixed Sample Set Assignment

We divided *O. mykiss* individuals in our genetic stock identification (GSI) analysis reference set in two different ways: (1) retaining all individuals sampled prior to dam removal with concordant physical and genetic population data and (2) retaining all individuals sampled prior to dam removal. The reference samples of the first sample set included individuals that were collected prior to dam removal and had concordant assignment between sampling location and DAPC population assignment (for K = 4). The reference samples of the second sample set included all individuals that were collected prior to dam removal, regardless of the concordance of assignment between sampling location and DAPC population assignment. We included all post-dam removal individuals in both mixed sample sets. However, the first mixed sample set also included individuals collected prior to dam removal not included in the reference set because their sampling location was not concordant with their DAPC population assignment.

Genetic stock identification analyses allow you to combine populations of fish from various sampling sites or collections into reporting units that are groups of fish that are genetically, geographically and from a management perspective similar (Moran and Anderson 2019; Seeb et al., 2007). RUBIAS takes information on reporting unit and collection site for analysis. Assignment of individuals to reporting units and collections for all GSI analyses followed the methods in the main text. However, individuals in the first reference sampling set that were sampled pre-dam removal but were not included in the reference sampling set due to discordance were assigned a mixture collection of “pre-dam removal” with reporting units also assigned as “NA”.

We chose to test both sample sets as our genetic data set included individuals that were sampled at all life history stages. As a result, sampling location information from juvenile fish may not have been representative of ultimate spawning location, which could bias the accuracy of our genetic stock identification panel. Furthermore, we wanted to account for any possible human error with labelling samples or any unaccounted for stocking events that may have resulted in unnatural fish translocations along the river.

### Appendix A.2. Selection of Loci to Be Used in GSI

Using Weir and Cockerham’s F_ST_ estimator (Weir and Cockerham 1984), we identified the top 100 SNPs with the largest pairwise F_ST_ between each pair of reference reporting units, with the exception of the SBLR. Pairwise comparisons with the SBLR were excluded as this location was located above an impassable barrier that was not hypothesized to produce any recolonizing steelhead. We also excluded loci on chromosome 5 (Omy5) to prevent overrepresentation of SNPs in high linkage disequilibrium within the double chromosomal inversion (Pearse et al., 2019). Instead, we retained twelve Omy5 SNPs that were identified in a previous study as informative for association with the anadromy phenotype (Micheletti et al., 2018; Pearse et al., 2019).

Since retaining individuals that were retained concordant population assignment to sampling location would most likely upwardly bias the accuracy of a GSI panel, we also tested the SNP set used for reference sample set one with reference sample set two. Therefore, in total, we conducted three GSI analyses using two sample sets and two SNP sets:

GSI analysis (i)   Sample set one + SNP set one;
GSI analysis (ii)   Sample set two + SNP set two;
GSI analysis (iii)   Sample set two + SNP set one.

Hereafter, we refer to each GSI analysis conducted on the SNP and sample combinations above as GSI analysis (i), GSI analysis (ii), and GSI analysis (iii).

### Appendix A.3. Self-Assignment Accuracy Assessment

We tested the accuracy of individual assignment to collections and reporting units based on our reference sample set using the leave-one-out approach implemented in the “self-assign” function in RUBIAS (Moran and Anderson 2019). Accuracy of collection assignment was assessed by calculating the average of the scaled likelihood of assignment of individuals back to their respective collection. The accuracy of the reporting units was calculated by summing the scaled likelihoods for each individual per collection and calculating the average across collections within each reporting unit. We also performed simulations to determine the expected accuracy for individual assignment back to their respective collections and reporting units. Using the “assess_reference_loo” function, we simulated 500 replicates for a mixture size of 120 individuals. The collections simulated had equal Dirichlet priors meaning that each simulation contained equal proportions of each collection for each reporting unit.

### Appendix A.4. GSI Mixture Analysis

We investigated whether steelhead recolonizing the Elwha River originated from outside the watershed using the Z-scores produced from individuals in the mixed sample set. Individual Z-scores were computed by taking the log-likelihood of membership to a reference collection based on the individual’s genotypes. Individual Z-scores for each fish in the reference sample set were also produced using the self-assign function. We then calculated the range of Z-scores for each reference collection and looked for individuals in the mixed sample set with Z-scores that fell out of the range of the reference collection. Individuals in the mixed sample set with Z-scores that fell outside of the reference collection’s range of Z-scores were removed from further analysis as we hypothesized that they may have been of non-Elwha ancestry or unsampled Elwha ancestry. We then calculated the relative proportion of reporting unit ancestry in each mixed sample set collection (steelhead life history cohort). Posterior density curves and 95% credible intervals for each reporting unit within each cohort mixture were created from 1000 iterations of the MCMC. Ancestry proportions for individuals sampled prior to dam removal, but not included in the reference, were similarly summarized.

**Table A1:** The accuracy of assignment of individuals in the reference collections used in GSI analysis (ii) were lower than those used in GSI analysis (i). Simulations performed on all 567 individuals sampled pre-dam removal using SNP set 2 reflected high accuracy of assignment on average across individuals in each reference collections back to their respective reporting units. The posterior likelihoods for reporting units’ assignment displayed above reflects the mean of the reporting unit scaled likelihoods of assignment based on collection membership within the reporting unit. The reporting unit scaled likelihood is calculated by taking the summation of the likelihoods of assigning a fish back to the inferred reporting unit. Posterior probability of assignment for Aldwell, Cat Creek and Whiskey Bend was low compared to GSI analysis (i).

Sampling Site	Reporting Unit	Inferred Reporting Unit			
		AD	ID	SBLR	BD
Chicago Camp	AD	**1.000**	0.000	0.000	0.000
Wilder	AD	**1.000**	0.000	0.000	0.000
Hayes	AD	**1.000**	0.000	0.000	0.000
Elkhorn	AD	**1.000**	0.000	0.000	0.000
Geyser	AD	**1.000**	0.000	0.000	0.000
Whiskey Bend	AD	**0.717**	0.283	0.000	0.000
Cat Creek	AD	**0.565**	0.387	0.000	0.048
Altaire	ID	0.000	**1.000**	0.000	0.000
Campground Creek	ID	0.030	**0.966**	0.000	0.004
Madison Creek	ID	0.000	**1.000**	0.000	0.000
Little River	ID	0.001	**0.996**	0.000	0.003
Indian Creek	ID	0.000	**1.000**	0.000	0.000
Aldwell	ID	0.613	**0.387**	0.000	0.000
South Branch Little River	SBLR	0.000	0.012	**0.942**	0.046
Elwha River	BD	0.005	0.058	0.000	**0.937**

**Table A2:** The accuracy of assignment of individuals in the reference collections used in GSI analysis (iii) were lower on average than those used in GSI analysis (i) or GSI analysis (ii). Simulations performed on all 567 individuals sampled pre-dam removal using SNP set 1 reflected lower accuracy of assignment on average across individuals in each reference collections back to their respective reporting units.

Sampling Site	Reporting Unit	Inferred Reporting Unit			
		AD	ID	SBLR	BD
Chicago Camp	AD	**1.000**	0.000	0.000	0.000
Wilder	AD	**1.000**	0.000	0.000	0.000
Hayes	AD	**1.000**	0.000	0.000	0.000
Elkhorn	AD	**1.000**	0.000	0.000	0.000
Geyser	AD	**0.950**	0.050	0.000	0.000
Whiskey Bend	AD	**0.349**	0.531	0.000	0.120
Cat Creek	AD	**0.452**	0.534	0.000	0.014
Altaire	ID	0.170	**0.830**	0.000	0.000
Campground Creek	ID	0.094	**0.906**	0.000	0.000
Madison Creek	ID	0.001	**0.999**	0.000	0.000
Little River	ID	0.020	**0.980**	0.000	0.000
Indian Creek	ID	0.000	**1.000**	0.000	0.000
Aldwell	ID	0.451	**0.549**	0.000	0.000
South Branch Little River	SBLR	0.004	0.031	**0.943**	0.022
Elwha River	BD	0.034	0.031	0.000	**0.934**

## References

[1] Haddad N.M., Brudvig L.A., Clobert J., Davies K.F., Gonzalez A., Holt R.D., Lovejoy T.E., Sexton J.O., Austin M.P., Collins C.D. (2015). Habitat fragmentation and its lasting impact on Earth’s ecosystems. Sci. Adv..

[2] Wu H., Chen J., Xu J., Zeng G., Sang L., Liu Q., Yin Z., Dai J., Yin D., Liang J. (2019). Effects of dam construction on biodiversity: A review. J. Clean. Prod..

[3] Liermann C.R., Nilsson C., Robertson J., Ng R.Y. (2012). Implications of Dam Obstruction for Global Freshwater Fish Diversity. Bioscience.

[4] Gosset C., Rives J., LaBonne J. (2006). Effect of habitat fragmentation on spawning migration of brown trout (*Salmo trutta* L.). Ecol. Freshw. Fish.

[5] Leclerc E., Mailhot Y., Mingelbier M., Bernatchez L. (2008). The landscape genetics of yellow perch (*Perca flavescens*) in a large fluvial ecosystem. Mol. Ecol..

[6] Winans G.A., Baker J., McHenry M., Ward L., Myers J. (2017). Genetic Characterization of Oncorhynchus mykiss Prior to Dam Removal with Implications for Recolonization of the Elwha River Watershed, Washington. Trans. Am. Fish. Soc..

[7] Winans G.A., Allen M.B., Baker J., Lesko E., Shrier F., Strobel B., Myers J. (2018). Dam trout: Genetic variability in Oncorhynchus mykiss above and below barriers in three Columbia River systems prior to restoring migrational access. PLoS ONE.

[8] Poff N.L., Olden J.D., Merritt D.M., Pepin D.M. (2007). Homogenization of regional river dynamics by dams and global biodiversity implications. Proc. Natl. Acad. Sci. USA.

[9] Beechie T., Moir H., Pess G. (2008). Hierarchical Physical Controls on Salmonid Spawning Location and Timing. Salmonid Spawning Habitat in Rivers: Physical Controls, Biological Responses, and Approaches to Remediation.

[10] Pearse D.E., Hayes S.A., Bond M.H., Hanson C.V., Anderson E.C., Macfarlane R.B., Garza J.C. (2009). Over the Falls? Rapid Evolution of Ecotypic Differentiation in Steelhead/Rainbow Trout (*Oncorhynchus mykiss*). J. Hered..

[11] Nehlsen W., Williams J.E., Lichatowich J.A. (1991). Pacific Salmon at the Crossroads—Stocks at Risk from California, Oregon, Idaho, and Washington. Fisheries.

[12] Ackiss A.S., Dang B.T., Bird C.E., Biesack E.E., Chheng P., Phounvisouk L., Vu Q.H., Uy S., Carpenter K.E. (2019). Cryptic Lineages and a Population Dammed to Incipient Extinction? Insights into the Genetic Structure of a Mekong River Catfish. J. Hered..

[13] Holecek D.E., Scarnecchia D.L., Miller S.E. (2012). Smoltification in an Impounded, Adfluvial Redband Trout Population Upstream from an Impassable Dam: Does It Persist?. Trans. Am. Fish. Soc..

[14] McClure M.M., Carlson S.M., Beechie T.J., Pess G.R., Jorgensen J.C., Sogard S.M., Sultan S.E., Holzer D.M., Travis J., Sanderson B.L. (2008). Evolutionary consequences of habitat loss for Pacific anadromous salmonids. Evol. Appl..

[15] Quinn T.P. (2005). The Behaviour and Ecology of Pacific Salmon and Trout.

[16] Behnke R.J. (2002). Trout and Salmon of North America.

[17] Moyle P.B. (2002). Inland Fishes of California: Revised and Expanded.

[18] Waples R.S., Zabel R.W., Scheuerell M.D., Sanderson B.L. (2008). Evolutionary responses by native species to major anthropogenic changes to their ecosystems: Pacific salmon in the Columbia River hydropower system. Mol. Ecol..

[19] Olsen J.B., Wuttig K., Fleming D., Kretschmer E.J., Wenburg J.K. (2006). Evidence of partial anadromy and resident-form dispersal bias on a fine scale in populations of Oncorhynchus mykiss. Conserv. Genet..

[20] Fraser D.J., Weir L.K., Bernatchez L., Hansen M.M., Taylor E.B. (2011). Extent and scale of local adaptation in salmonid fishes: Review and meta-analysis. Heredity.

[21] Katz J., Moyle P.B., Quiñones R.M., Israel J., Purdy S. (2012). Impending extinction of salmon, steelhead, and trout (Salmonidae) in California. Environ. Biol. Fishes.

[22] Kendall N.W., McMillan J.R., Sloat M.R., Buehrens T.W., Quinn T.P., Pess G.R., Kuzishchin K.V., McClure M.M., Zabel R.W., Bradford M. (2015). Anadromy and residency in steelhead and rainbow trout (*Oncorhynchus mykiss*): A review of the processes and patterns. Can. J. Fish. Aquat. Sci..

[23] Hoar W.S. (1988). The Physiology of Smolting Salmonids. Fish Physiology.

[24] Nichols K.M., Edo A.F., Wheeler P.A., Thorgaard G.H. (2008). The Genetic Basis of Smoltification-Related Traits in Oncorhynchus mykiss. Genetics.

[25] Thrower F.P., Hard J.J., Joyce J.E. (2004). Genetic architecture of growth and early life history transitions in anadromous and derived freshwater populations of steelhead. J. Fish Biol..

[26] Hecht B.C., Thrower F.P., Hale M.C., Miller M.R., Nichols K.M. (2012). Genetic Architecture of Migration-Related Traits in Rainbow and Steelhead Trout, *Oncorhynchus mykiss*. G3 Genes Genomes Genet..

[27] Narum S.R., Zendt J.S., Graves D., Sharp W.R. (2008). Influence of landscape on resident and anadromous life history types of Oncorhynchus mykiss. Can. J. Fish. Aquat. Sci..

[28] McMillan J.R., Dunham J.B., Reeves G.H., Mills J.S., Jordan C.E. (2011). Individual condition and stream temperature influence early maturation of rainbow and steelhead trout, Oncorhynchus mykiss. Environ. Biol. Fishes.

[29] Hecht B.C., Valle M.E., Thrower F.P., Nichols K.M. (2014). Divergence in Expression of Candidate Genes for the Smoltification Process Between Juvenile Resident Rainbow and Anadromous Steelhead Trout. Mar. Biotechnol..

[30] McKinney G.J., Hale M.C., Goetz G., Gribskov M., Thrower F.P., Nichols K.M. (2015). Ontogenetic changes in embryonic and brain gene expression in progeny produced from migratory and resident Oncorhynchus mykiss. Mol. Ecol..

[31] Narum S.R., Contor C., Powell M.S. (2004). Genetic divergence of sympatric resident and anadromous forms of Oncorhynchus mykiss in the Walla Walla River, U.S.A. J. Fish Biol..

[32] Heath A.C., Martin N.G., Montgomery G.W., Goddard M.E., Visscher P.M. (2010). Common SNPs explain a large proportion of the heritability for human height. Nat. Genet..

[33] Clemento A.J., Anderson E.C., Boughton D., Girman D., Garza J.C. (2009). Population genetic structure and ancestry of Oncorhynchus mykiss populations above and below dams in south-central California. Conserv. Genet..

[34] Docker M.F., Dale A., Heath D.D. (2003). Erosion of interspecific reproductive barriers resulting from hatchery supplementation of rainbow trout sympatric with cutthroat trout. Mol. Ecol..

[35] Deiner K., Garza J.C., Coey R., Girman D. (2007). Population structure and genetic diversity of trout (*Oncorhynchus mykiss*) above and below natural and man-made barriers in the Russian River, California. Conserv. Genet..

[36] Pearse D.E., Miller M.R., Abadía-Cardoso A., Garza J.C. (2014). Rapid parallel evolution of standing variation in a single, complex, genomic region is associated with life history in steelhead/rainbow trout. Proc. R. Soc. B Biol. Sci..

[37] Leitwein M., Garza J.C., Pearse D.E. (2017). Ancestry and adaptive evolution of anadromous, resident, and adfluvial rainbow trout (*Oncorhynchus mykiss*) in the San Francisco bay area: Application of adaptive genomic variation to conservation in a highly impacted landscape. Evol. Appl..

[38] Micheletti S.J., Hess J.E., Zendt J.S., Narum S.R. (2018). Selection at a genomic region of major effect is responsible for evolution of complex life histories in anadromous steelhead. BMC Evol. Biol..

[39] Pearse D.E., Barson N.J., Nome T., Gao G., Campbell M.A., Abadía-Cardoso A., Anderson E.C., Rundio D.E., Williams T.H., Naish K.A. (2019). Sex-dependent dominance maintains migration supergene in rainbow trout. Nat. Ecol. Evol..

[40] Hecht B.C., Campbell N.R., Holecek D.E., Narum S.R. (2013). Genome-wide association reveals genetic basis for the propensity to migrate in wild populations of rainbow and steelhead trout. Mol. Ecol..

[41] Arostegui M.C., Quinn T.P., Seeb L.W., Seeb J.E., McKinney G.J. (2019). Retention of a chromosomal inversion from an anadromous ancestor provides the genetic basis for alternative freshwater ecotypes in rainbow trout. Mol. Ecol..

[42] Weinstein S.Y., Thrower F.P., Nichols K.M., Hale M.C. (2019). A large-scale chromosomal inversion is not associated with life history development in rainbow trout from Southeast Alaska. PLoS ONE.

[43] Dodson J.J., Aubin-Horth N., Theriault V., Paez D.J. (2013). The evolutionary ecology of alternative migratory tactics in salmonid fishes. Biol. Rev..

[44] Hess J.E., Zendt J.S., Matala A.R., Narum S.R. (2016). Genetic basis of adult migration timing in anadromous steelhead discovered through multivariate association testing. Proc. R. Soc. B Biol. Sci..

[45] Prince D.J., O’Rourke S.M., Thompson T.Q., Ali O.A., Lyman H.S., Saglam I.K., Hotaling T.J., Spidle A.P., Miller M.R. (2017). The evolutionary basis of premature migration in Pacific salmon highlights the utility of genomics for informing conservation. Sci. Adv..

[46] Micheletti S.J., Matala A.R., Matala A.P., Narum S.R. (2018). Landscape features along migratory routes influence adaptive genomic variation in anadromous steelhead (*Oncorhynchus mykiss*). Mol. Ecol..

[47] Thompson T.Q., Bellinger M.R., O’Rourke S.M., Prince D.J., Stevenson A.E., Rodrigues A.T., Sloat M.R., Speller C.F., Yang D.Y., Butler V.L. (2019). Anthropogenic habitat alteration leads to rapid loss of adaptive variation and restoration potential in wild salmon populations. Proc. Natl. Acad. Sci. USA.

[48] Thompson N.F., Anderson E.C., Clemento A.J., Campbell M.A., Pearse D.E., Hearsey J.W., Kinziger A.P., Garza J.C. (2020). A complex phenotype in salmon controlled by a simple change in migratory timing. Science.

[49] Willis S.C., Hess J.E., Fryer J.K., Whiteaker J.M., Brun C., Gerstenberger R., Narum S.R. (2020). Steelhead (*Oncorhynchus mykiss*) lineages and sexes show variable patterns of association of adult migration timing and age-at-maturity traits with two genomic regions. Evol. Appl..

[50] Collins E.E., Hargrove J.S., Delomas T.A., Narum S.R. (2020). Distribution of genetic variation underlying adult migration timing in steelhead of the Columbia River basin. Ecol. Evol..

[51] Lindley S.T., Schick R.S., Agrawal A., Goslin M., Pearson T.E., Mora E., Anderson J.J., May B., Greene S., Hanson C. (2006). Historical Population Structure of Central Valley Steelhead and its Alteration by Dams. San Franc. Estuary Watershed Sci..

[52] Blanchet S., Prunier J.G., Paz-Vinas I., Saint-Pé K., Rey O., Raffard A., Mathieu-Bégné E., Loot G., Fourtune L., Dubut V. (2020). A river runs through it: The causes, consequences, and management of intraspecific diversity in river networks. Evol. Appl..

[53] Loomis J.B. (1996). Measuring the Economic Benefits of Removing Dams and Restoring the Elwha River: Results of a Contingent Valuation Survey. Water Resour. Res..

[54] Wunderlich R.C., Winter B.D., Meyer J.H. (1994). Restoration of the Elwha River Ecosystem. Fisheries.

[55] Duda J.J., Freilich J.E., Schreiner E.G. (2008). Baseline Studies in the Elwha River Ecosystem Prior to Dam Removal: Introduction to the Special Issue. Northwest Sci..

[56] Winans G.A., McHenry M.L., Baker J., Elz A., Goodbla A., Iwamoto E., Kuligowski D., Miller K.M., Small M.P., Spruell P. (2008). Genetic Inventory of Anadromous Pacific Salmonids of the Elwha River Prior to Dam Removal. Northwest Sci..

[57] Pess G.R., McHenry M.L., Beechie T.J., Davies J. (2008). Biological Impacts of the Elwha River Dams and Potential Salmonid Responses to Dam Removal. Northwest Sci..

[58] Brenkman S.J., Pess G.R., Torgersen C.E., Kloehn K.K., Duda J.J., Corbett S.C. (2008). Predicting Recolonization Patterns and Interactions Between Potamodromous and Anadromous Salmonids in Response to Dam Removal in the Elwha River, Washington State, USA. Northwest Sci..

[59] Brenkman S.J., Mumford S.L., House M., Patterson C. (2008). Establishing Baseline Information on the Geographic Distribution of Fish Pathogens Endemic in Pacific Salmonids Prior to Dam Removal and Subsequent Recolonization by Anadromous Fish in the Elwha River, Washington. Northwest Sci..

[60] Brenkman S.J., Duda J.J., Torgersen C.E., Welty E., Pess G.R., Peters R., McHenry M.L. (2012). A riverscape perspective of Pacific salmonids and aquatic habitats prior to large-scale dam removal in the Elwha River, Washington, USA. Fish. Manag. Ecol..

[61] Pess G.R., McHenry M.L., Denton K., Anderson J.H., Liermann M.C., Peters R.J., Brenkman S., Bennett T.R. (2020). Initial response of Chinook salmon (*Oncorhynchus tshawytscha*) and steelhead (*Oncorhynchus mykiss*) to removal of two dams on the Elwha River, Washington State, U.S.A. Can. J. Fish. Aquat. Sci..

[62] Ali O.A., O’Rourke S.M., Amish S.J., Meek M.H., Luikart G., Jeffres C., Miller M.R. (2016). RAD Capture (Rapture): Flexible and Efficient Sequence-Based Genotyping. Genetics.

[63] Paris J.R., Stevens J.R., Catchen J.M. (2017). Lost in parameter space: A road map for stacks. Methods Ecol. Evol..

[64] Catchen J., Hohenlohe P.A., Bassham S., Amores A., Cresko W.A. (2013). Stacks: An analysis tool set for population genomics. Mol. Ecol..

[65] Li H., Durbin R. (2009). Making the Leap: Maq to BWA. Mass Genom..

[66] Li H. (2011). A statistical framework for SNP calling, mutation discovery, association mapping and population genetical parameter estimation from sequencing data. Bioinformatics.

[67] Danecek P., Auton A., Abecasis G., Albers C.A., Banks E., Depristo M.A., Handsaker R.E., Lunter G., Marth G.T., Sherry S.T. (2011). The variant call format and VCFtools. Bioinformatics.

[68] Glasauer S.M., Neuhauss S.C. (2014). Whole-genome duplication in teleost fishes and its evolutionary consequences. Mol. Genet. Genom..

[69] McKinney G.J., Waples R.K., Seeb L.W., Seeb J.E. (2017). Paralogs are revealed by proportion of heterozygotes and deviations in read ratios in genotyping-by-sequencing data from natural populations. Mol. Ecol. Resour..

[70] Jombart T. (2008). adegenet: A R package for the multivariate analysis of genetic markers. Bioinformatics.

[71] Jombart T., Ahmed I. (2011). adegenet 1.3-1: New tools for the analysis of genome-wide SNP data. Bioinformatics.

[72] Raj A., Stephens M., Pritchard J.K. (2014). fastSTRUCTURE: Variational Inference of Population Structure in Large SNP Data Sets. Genetics.

[73] Weir B.S., Cockerham C.C. (1984). Estimating F-Statistics for the Analysis of Population Structure. Evolution.

[74] Tajima F. (1989). Statistical Method for Testing the Neutral Mutation Hypothesis by DNA Polymorphism. Genetics.

[75] Nei M., Li W.H. (1979). Mathematical model for studying genetic variation in terms of restriction endonucleases. Proc. Natl. Acad. Sci. USA.

[76] Do C., Waples R.S., Peel D., Macbeth G.M., Tillett B.J., Ovenden J.R. (2014). NeEstimator v2: Re-implementation of software for the estimation of contemporary effective population size (Ne) from genetic data. Mol. Ecol. Resour..

[77] Puth M.T., Neuhäuser M., Ruxton G.D. (2015). On the variety of methods for calculating confidence intervals by bootstrapping. J. Anim. Ecol..

[78] Moran B.M., Anderson E.C. (2019). Bayesian inference from the conditional genetic stock identification model. Can. J. Fish. Aquat. Sci..

[79] Anderson E.C., Waples R.S., Kalinowski S.T. (2008). An improved method for predicting the accuracy of genetic stock identification. Can. J. Fish. Aquat. Sci..

[80] Miller M.R., Brunelli J.P., Wheeler P.A., Liu S., Rexroad C.E., Palti Y., Doe C.Q., Thorgaard G.H. (2012). A conserved haplotype controls parallel adaptation in geographically distant salmonid populations. Mol. Ecol..

[81] Bates D., Mächler M., Bolker B., Walker S. (2015). Fitting linear mixed-effects models using lme4. J. Stat. Softw..

[82] Rousset F. (2008). genepop’007: A complete re-implementation of the genepop software for Windows and Linux. Mol. Ecol. Resour..

[83] Abadía-Cardoso A., Pearse D.E., Jacobson S., Marshall J., Dalrymple D., Kawasaki F., Ruiz-Campos G., Garza J.C. (2016). Population genetic structure and ancestry of steelhead/rainbow trout (*Oncorhynchus mykiss*) at the extreme southern edge of their range in North America. Conserv. Genet..

[84] Phelps S.R., Hiss J.M., Peters R.J. (1999). Genetic Relationships of Elwha River Oncorhynchus mykiss to Hatchery-Origin Rainbow Trout and Washington Steelhead.

[85] Robison E.G., Mirati A., Allen M. (2000). Oregon Road/Stream Crossing Restoration Guide: Spring 1999.

[86] Samarasin P., Shuter B.J., Rodd F.H. (2017). After 100 years: Hydroelectric dam-induced life-history divergence and population genetic changes in sockeye salmon (*Oncorhynchus nerka*). Conserv. Genet..

[87] Limborg M.T., Blankenship S.M., Young S.F., Utter F.M., Seeb L.W., Hansen M.H., Seeb J.E. (2012). Signatures of natural selection among lineages and habitats inOncorhynchus mykiss. Ecol. Evol..

[88] Busby P.J., Wainright T.C., Bryant G.J., Lierheimer L.J., Waples R.S., Waknitz F.W., Lagomarsino I.V. (1996). Status Review of West Coast Steelhead from Washington, Idaho, Oregon, and California.

[89] Peacock M.M., Gustin M.S., Kirchoff V.S., Robinson M.L., Hekkala E., Pizzarro-Barraza C., Loux T. (2016). Native fishes in the Truckee River: Are in-stream structures and patterns of population genetic structure related?. Sci. Total Environ..

[90] Kelson S.J., Miller M.R., Thompson T.Q., O’Rourke S.M., Carlson S.M. (2020). Temporal dynamics of migration-linked genetic variation are driven by streamflows and riverscape permeability. Mol. Ecol..

[91] Hiss J.M., Wunderlich R.C. (1994). Salmonid Availability and Migration in the Middle Elwha River System. Miscellaneous Report.

[92] Keefer M.L., Caudill C.C. (2014). Homing and straying by anadromous salmonids: A review of mechanisms and rates. Rev. Fish Biol. Fish..

[93] Hendry A.P., Castric V., Kinnison M.T., Quinn T.P., Hendry A., Stearns S. (2004). The evolution of philopatry and dispersal. Evolution Illuminated: Salmon and Their Relatives.

[94] Ostberg C.O., Slatton S.L., Rodriguez R.J. (2004). Spatial partitioning and asymmetric hybridization among sympatric coastal steelhead trout (*Oncorhynchus mykiss* irideus), coastal cutthroat trout (*O. clarki clarki*) and interspecific hybrids. Mol. Ecol..

[95] Kawecki T.J., Ebert D. (2004). Conceptual issues in local adaptation. Ecol. Lett..

[96] Davis C.D., Epps C.W., Flitcroft R.L., Banks M.A. (2018). Refining and defining riverscape genetics: How rivers influence population genetic structure. Wiley Interdiscip. Rev. Water.

[97] Brannon E.L., Powell M.S., Quinn T.P., Talbot A. (2010). Population Structure of Columbia River Basin Chinook Salmon and Steelhead Trout. Rev. Fish. Sci..

[98] Luikart G., Ryman N., Tallmon D.A., Schwartz M.K., Allendorf F.W. (2010). Estimation of census and effective population sizes: The increasing usefulness of DNA-based approaches. Conserv. Genet..

