# Correction: Fraik et al. The Impacts of Dam Construction and Removal on the Genetics of Recovering Steelhead (*Oncorhynchus mykiss*) Populations across the Elwha River Watershed. *Genes* 2021, *12*, 89

**DOI:** 10.3390/genes13091638

**Published:** 2022-09-13

**Authors:** Alexandra K. Fraik, John R. McMillan, Martin Liermann, Todd Bennett, Michael L. McHenry, Garrett J. McKinney, Abigail H. Wells, Gary Winans, Joanna L. Kelley, George R. Pess, Krista M. Nichols

**Affiliations:** 1 School of Biological Sciences, Washington State University, Pullman, WA 99164, USA; 2 Trout Unlimited, 1777 N. Kent Street, Suite 100, Arlington, VA 22209, USA; 3 Northwest Fisheries Science Center, National Marine Fisheries Service, National Oceanic and Atmospheric Administration, 2725 Montlake Boulevard East, Seattle, WA 98112, USA; 4 Lower Elwha Klallam Tribe Natural Resources, 760 Stratton Road, Port Angeles, WA 98363, USA; 5 Lynker Technologies, in Support of the Conservation Biology Division, Northwest Fisheries Science Center, National Marine Fisheries Service, National Oceanic and Atmospheric Administration, 2725 Montlake Boulevard East, Seattle, WA 98112, USA

## 1. Modified Main Text 

In the original publication [1], the main text (including figures, tables and appendix figures) of the manuscript need to be revised.

### 1.1. Introduction

Paragraph 6: Change due to metadata coding errors.

In this study, we genotyped 71,320 SNPs from 567 samples of both steelhead and resident rainbow trout collected prior to dam removal and from 558 steelhead post-dam removal to investigate how steelhead are recolonizing the river. 

To

In this study, we genotyped 71,320 SNPs from 567 samples of both steelhead and resident rainbow trout collected prior to dam removal and from 556 steelhead post-dam removal to investigate how steelhead are recolonizing the river. 

### 1.2. Methods

Section 2.1, Paragraph 3: Change due to metadata coding errors.

Following the removal of the second dam, the upriver Glines Canyon Dam (2014), the lower (below both former dams) and middle portions (between both former dams) of the Elwha River were mainly sampled with no samples collected from the upper portion (formerly above the dams). Juvenile smolts were sampled in rotary screw traps from three sampling sites: Little River (river kilometer (rkm) 0.2), Indian Creek (rkm 0.6), and on the main stem Elwha River (0.3 and 3.3 in 2014–2018 and rkm 4.0 in 2019) [61].

To

Following the removal of the second dam, the upriver Glines Canyon Dam (2014), the lower (below both former dams) and middle portions (between both former dams) of the Elwha River were mainly sampled, with only three samples collected from the upper portion (formerly above the dams). Juvenile smolts were sampled in rotary screw traps from three sampling sites in 2016 and 2017: Little River (river kilometer (rkm) 0.2), Indian Creek (rkm 0.6), and on the main stem Elwha River (0.3–3.3 rkm) [61].

Section 2.1, Paragraph 3: Change due to metadata coding errors.

Adult steelhead were sampled during weekly or bi-weekly in-river tangle net sampling conducted at nine different sites in the lower, and to a lesser extent, middle portion of the watershed [61].

To

Adult steelhead were sampled during weekly or bi-weekly in-river tangle net sampling conducted at ten different sites in the lower, and to a lesser extent, upper and middle portions of the watershed [61].

Section 2.1, Paragraph 5: Added four sentences to the end of this section paragraph to address questions raised about hatchery management practices on the Elwha River. 

Adult steelhead sampled post-dam removal included fish that were produced from naturally spawning adults as well as fish that originated from the Lower Elwha Klallam’s Tribe integrated hatchery program (Winans et al., 2017). This program used eyed eggs and trout fry from naturally spawning, native steelhead in the Lower portions of the Elwha River prior to dam removal (2005–2011) from below the lower Elwha River Dam (LEKT 2012). Samples from many of these naturally spawning parents were included in the below dam, prior to dam removal, collections included in this study, as well as previous genetic studies (Winans et al., 2017). In total, 209 steelhead sampled post-dam removal were identified as hatchery origin, 259 were of natural origin and 88 were of unknown origin. 

Section 2.3, Paragraph 1: Change due to metadata coding errors.

Post-filtering, we retained 1125 individual *Oncorhynchus mykiss* (567 individuals from 15 sampling sites pre-dam removal and 558 individuals from four sampling sites post-dam removal) and 71,320 SNPs (Table 1).

To

Post-filtering, we retained 1125 individual *Oncorhynchus mykiss* (567 individuals from 15 sampling sites pre-dam removal and 556 individuals from five sampling sites post-dam removal) and 71,320 SNPs (Table 1).

### 1.3. Results

Section 3.1, Paragraph 1: Change due to metadata coding errors.

After initial quality filtering of RADseq data, an average of ~1.5 million reads per individual were retained; removing PCR clones retained 961,682 reads for read mapping to the *O. mykiss* genome (Supplementary Table S1). We retained 1125 of the original 1334 individuals after filtering out individuals with high amounts of missing data, 567 of which were collected prior to dam removal and 558 steelhead following dam removal (Table 1). Reads mapped at an average rate of 97.3% per retained individual (Supplementary Table S1). 

To

After initial quality filtering of RADseq data, an average of ~1.5 million reads per individual were retained; removing PCR clones retained 960,812 reads for read mapping to the *O. mykiss* genome (Supplementary Table S1). We retained 1123 of the original 1334 individuals after filtering out individuals with high amounts of missing data, 567 of which were collected prior to dam removal and 556 steelhead following dam removal (Table 1). Reads mapped at an average rate of 97.3% per retained individual (Supplementary Table S1). 

Section 3.2, Paragraph 1: Change due to coding and coloring error in the script to generate the PCA plots.

There were no discernible clustering patterns in the PCA related to population, sampling site or migratory phenotype when including all loci sequenced among all individuals prior to dam removal (Supplementary Figure S1a) or post-dam removal (Figure S1b). 

To

There were some clustering patterns in the PCA related to population, but not to sampling site or migratory phenotype when including all loci sequenced among all individuals prior to dam removal (Supplementary Figure S1a) or post-dam removal (Figure S1b). 

Section 3.3, Paragraph 2: Change due to metadata coding errors and to address concerns about the impact of the hatchery management practices on the Elwha River on our findings.

Post-dam removal, however, we did not see any clear increase in genetic structure among populations or life history cohorts between genetic structure plots for K = 3 (Supplementary Figures S4c,d and S5d) compared to K = 2 (Figure 2b and Figure 3c).

To

Post-dam removal, however, we did not see any clear increase in genetic structure among populations, life history cohorts or hatchery origin status between genetic structure plots for K = 3 (Supplementary Figures S4c,d and S5d) compared to K = 2 (Figures 2b and 3c). 

Section 3.5.1, Paragraph 1: Change due to metadata coding errors.

The mixed sample set included the 558 steelhead samples collected post-dam removal and the 88 individuals collected prior to dam removal, but not included in the reference set. 

To

The mixed sample set included the 556 steelhead samples collected post-dam removal and the 88 individuals collected prior to dam removal, but not included in the reference set. 

Section 3.5.3, Paragraph 2: Change due to metadata coding errors and subsequent GSI reanalysis.

After removing the three individuals with low log-likelihood values (Supplementary Table S4), 85 samples were retained as mixture samples in our “pre-dam removal” life history cohort for GSI (Supplementary Table S5).

To

After removing the three individuals with low log-likelihood values (Supplementary Table S4), 86 samples were retained as mixture samples in our “pre-dam removal” life history cohort for GSI (Supplementary Table S5).

### 1.4. Discussion

Section 4.3, Paragraph 1: Change in result due to rerunning GSI analyses.

We observed no contribution of genetic ancestry from the South Branch of the Little River reference population to the returning steelhead, concordant with our expectations.

To

We observed minimal contributions of genetic ancestry from the South Branch of the Little River reference population to the returning steelhead, concordant with our expectations.

Section 4.3, Paragraph 2: Change a numeric typo that was never resolved.

Although adult steelhead cohorts lacked variation in GSI assignment of inferred population, out-migrating smolt cohorts (2017–2018) had high proportions of above- and in-between-dam genetic ancestries (Supplementary Figure S8). 

To

Although adult steelhead cohorts lacked variation in GSI assignment of inferred population, out-migrating smolt cohorts (2016–2017) had high proportions of above- and in-between-dam genetic ancestries (Supplementary Figure S8).

Section 4.3, Paragraph 3: Added discussion sentence to address questions raised about the impact of the hatchery management practices in the Elwha River on our findings, and to correct the numbers of non-Elwha fish with corrections to the metadata.

Across all of our GSI analyses, we did not detect more than six fish that appeared to be of non-Elwha origin (Supplementary Table S4). As we hypothesized initially, some recolonizing steelhead may have strayed from nearby watersheds. While steelhead and other anadromous salmonids are known to have return to their natal sites at high rates [92], they have been observed to exhibit density-dependent straying [93]. Another possibility is that there may have been undocumented stocking or translocations of fish from nearby watersheds into the upper or middle portions of the Elwha River. Stocking of *O. mykiss* in high-elevation lakes above-dam and in-between-dam high-elevation lakes were recorded on the Elwha River prior to dam removal until 2001 [59]. Goldendale Hatchery trout sourced from California were also supplemented to an in-between-dam location, Lake Sutherland, from 1995 until 2012 [6]. Movement of hatchery stock from this lake into Indian Creek and other in-between-dam tributaries was prevented by a rotating screen. While we did not include samples from any locations that were known to have been supplemented recently, we cannot necessarily rule out the possibility that fish were translocated by humans, animals or highwater flows. 

To

Across all of our GSI analyses, we did not detect more than five fish that appeared to be of non-Elwha origin (Supplementary Table S4). As we hypothesized initially, some recolonizing steelhead may have strayed from nearby watersheds. Although steelhead and other anadromous salmonids are known to return to their natal sites at high rates [92], they have been observed to exhibit density-dependent straying [93]. Another possibility is that there may have been undocumented stocking or translocations of fish from nearby watersheds into the upper or middle portions of the Elwha River. Stocking of *O. mykiss* in high-elevation lakes above-dam and in-between-dam high-elevation lakes was recorded on the Elwha River prior to dam removal until 2001 [59]. Goldendale Hatchery trout sourced from California were also supplemented to an in-between-dam location, Lake Sutherland, from 1995 until 2012 [6]. Movement of hatchery stock from this lake into Indian Creek and other in-between-dam tributaries was prevented by a rotating screen. Additionally, while the Chambers Creek hatchery program ceased in 2011, a native broodstock steelhead hatchery program began in 2005 (releases began in 2006) on the Lower Elwha and continues to release fish. However, both previous work and our analyses have not identified significant impacts of the Chambers Creek program nor the current hatchery program on the genetics of the native steelhead population (Winans et al. 2017; unpublished data). Although we did not include samples from any locations that were known to have been supplemented recently, we cannot necessarily rule out the possibility that fish were translocated by humans, animals or highwater flows.

## 2. Modified Main Text Figures

Original Figure 3:

**Figure 3:**
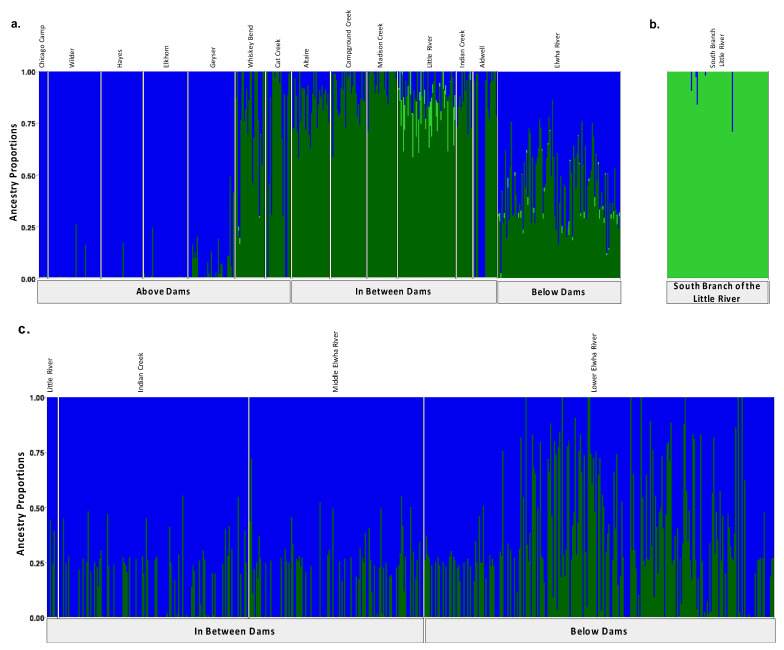
Population assignments computed by fastSTRUCTURE for samples collected (**a**,**b**) prior to dam removal supported three distinct genetic clusters (K = 3). (**a**) High levels of genetic structure were observed among populations split by man-made barriers such as dams (**b**) as well as natural barrier like the falls that separate the South Branch of the Little River from the Little River. (**c**) Post-dam removal, we detected two genetic clusters with increasing levels of admixture. Each vertical bar represents a single individual sampled at one of the sampling sites labelled across the top x axis which are organized from up-river to down river and divided by anadromous barrier location which are labelled on the bottom x axis. Each color represents a distinct genetic cluster.

Modified Figure 3: Updated plot, rearranging individuals with corrected metadata.

**Figure 3:**
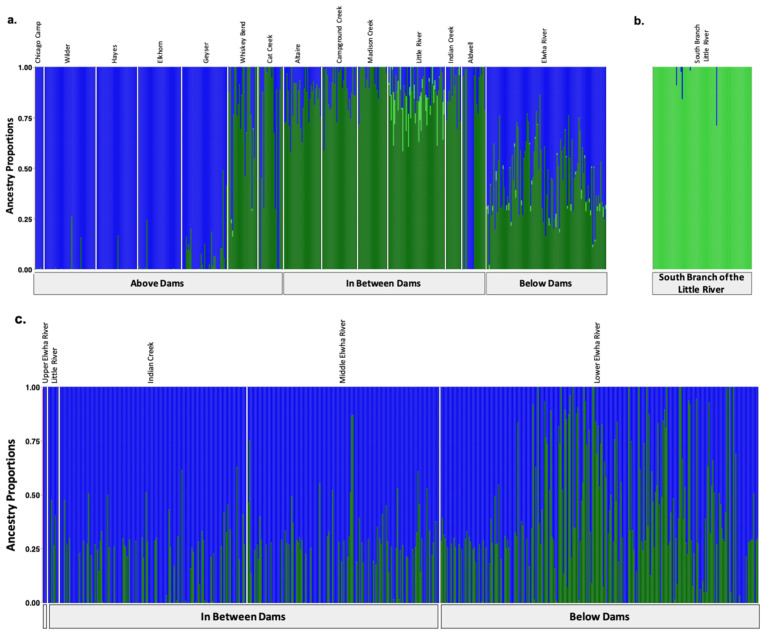
Population assignments computed by fastSTRUCTURE for samples collected (**a**,**b**) prior to dam removal supported three distinct genetic clusters (K = 3). (**a**) High levels of genetic structure were observed among populations split by man-made barriers such as dams (**b**) as well as natural barriers, such as the waterfalls that separate the South Branch of the Little River from the Little River. (**c**) Post-dam removal, we detected two genetic clusters with increasing levels of admixture. Each colored vertical bar represents a single individual sampled at one of the sampling sites labelled across the top x axis, organized from up-river to down-river and divided by anadromous barrier location, as labelled on the bottom x axis. The left-most samples in the plot, denoted by an unlabeled, empty gray box, include samples from above the dams, furthest up-river. Each color represents a distinct genetic cluster.

Original Figure 4:

**Figure 4:**
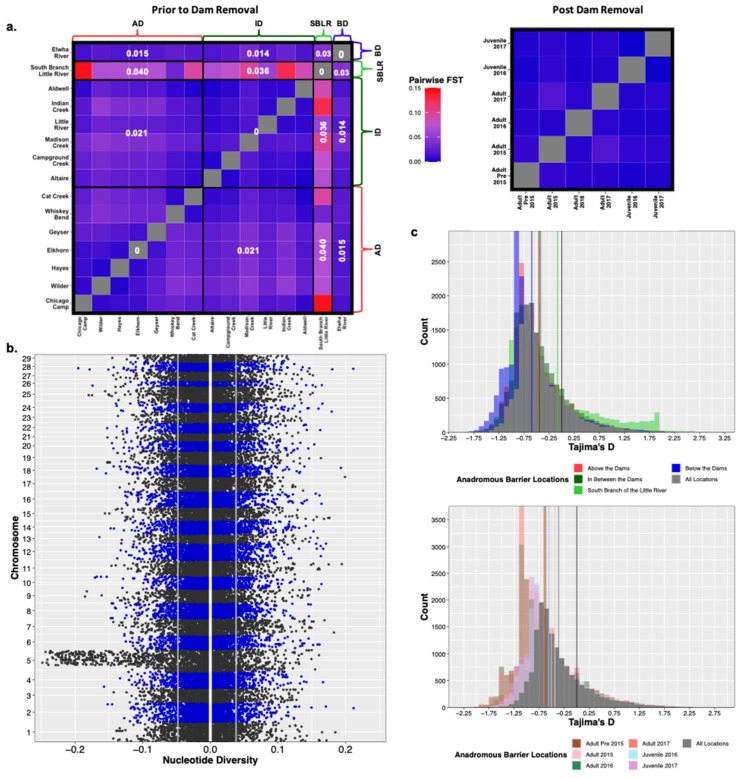
Prior to dam removal we saw genetic differentiation (FST) among populations separated by anadromous barriers, but little temporal differentiation among life history cohorts sampled post-dam removal (**a**). We saw a significant increase in nucleotide diversity (π) at Omy5 post-dam removal, but not genome wide (**b**). The white vertical line is at zero, the two grey lines represent the upper and lower bounds of bootstrapped confidence intervals. There were statistically insignificant increases in estimates of Tajima’s D temporally and among life history cohorts sampled post-dam removal (**c**).

Modified Figure 4: Updated plot, accounting for individuals with corrected metadata.

**Figure 4:**
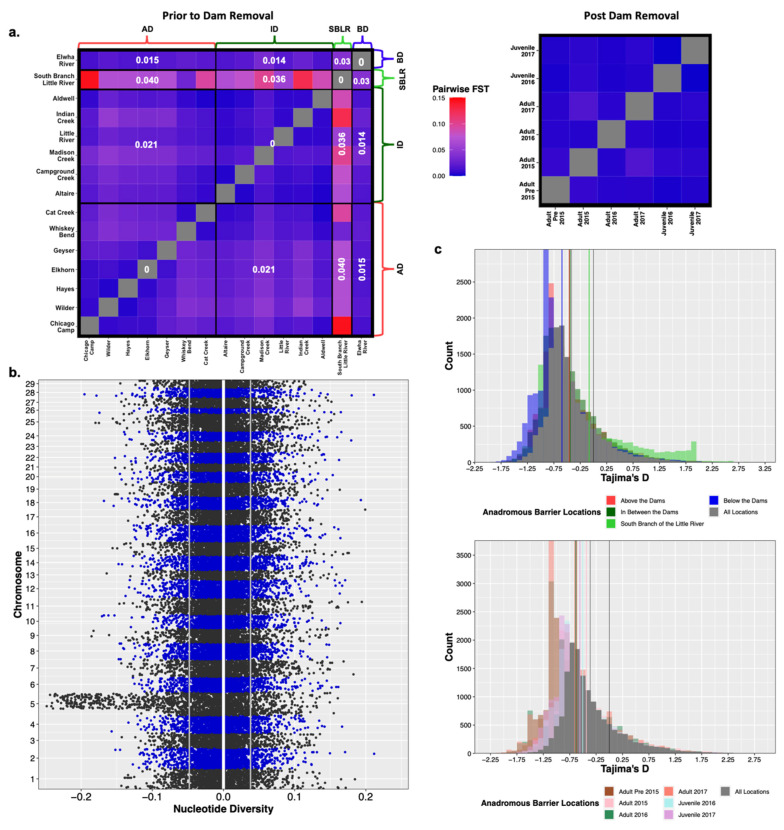
Prior to dam removal we saw genetic differentiation (FST) among populations separated by anadromous barriers, but little temporal differentiation among life history cohorts sampled post-dam removal (**a**). We saw a significant increase in nucleotide diversity (π) at Omy5 post-dam removal, but not genome wide (**b**). The white vertical line is at zero, the two grey lines represent the upper and lower bounds of bootstrapped confidence intervals. There were statistically insignificant increases in estimates of Tajima’s D temporally and among life history cohorts sampled post-dam removal (**c**).

Original Figure 5:

**Figure 5:**
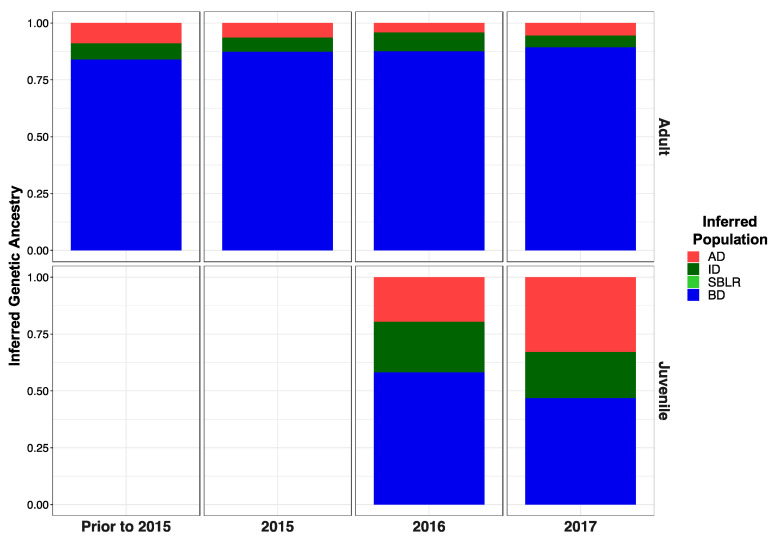
Inferred proportion of genetic ancestry from each of the four reference populations across recolonizing adult steelhead and juvenile smolt life history cohorts. Colors are representative of the inferred population. The bottom x axis divides life history cohorts temporally and the right y axis divides them by life history stage. We included the South Branch of the Little River (SBLR) as a control because we did not expect to see assignment to that population.

Modified Figure 5: Updated plot, rearranging individuals with corrected metadata.

**Figure 5:**
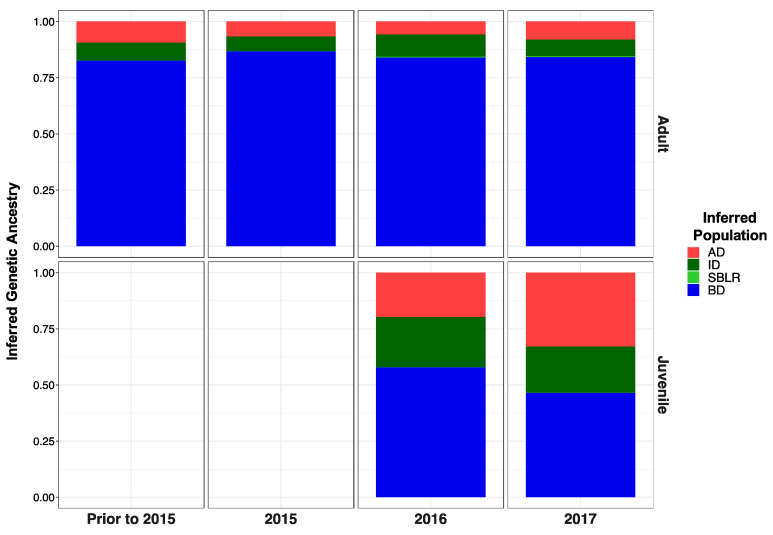
Inferred proportion of genetic ancestry from each of the four reference populations across recolonizing adult steelhead and juvenile smolt life history cohorts. Colors are representative of the inferred population. The bottom x axis divides life history cohorts temporally and the right y axis divides them by life history stage. We included the South Branch of the Little River (SBLR) as a control because we did not expect to see assignment to that population.

Original Figure 7:

**Figure 7:**
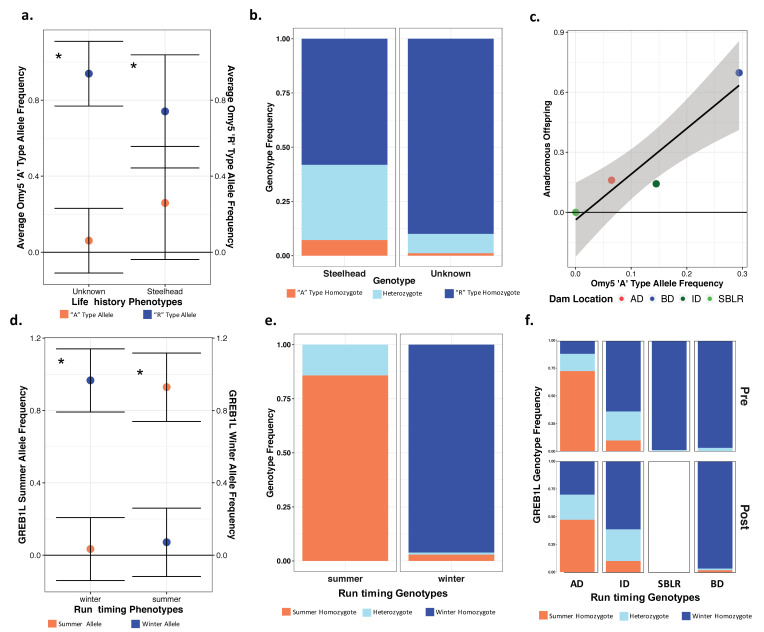
There were significant differences in the allelic and genotypic frequencies of the Omy5 (**a**–**c**) and the GREB1L variants (**d**–**f**) among populations and life histories. There were significant differences in the mean allele frequencies of the Omy5 locus allele frequencies between steelhead and unknown forms (**a**) and the frequency of the Omy5 “A” type allele frequency significantly explained life history phenotype (**b**). We also observed a strong correlation between the number of anadromous individuals from each population and the frequency of the “A” allele (**c**). The mean frequency of the GREB1L summer allele also varied significantly between summer- and winter-run steelhead (**d**), with the frequency of the homozygous summer genotype significantly explaining run timing (**e**), and inferred, ancestral population explaining a significant proportion of the variation in genotype both prior to and post-dam removal (**f**). Asterisks (**a**,**c**) indicate statistical significance (α = 0.01) (*).

Modified Figure 7: Updated plot, accounting for individuals with corrected metadata.

**Figure 7:**
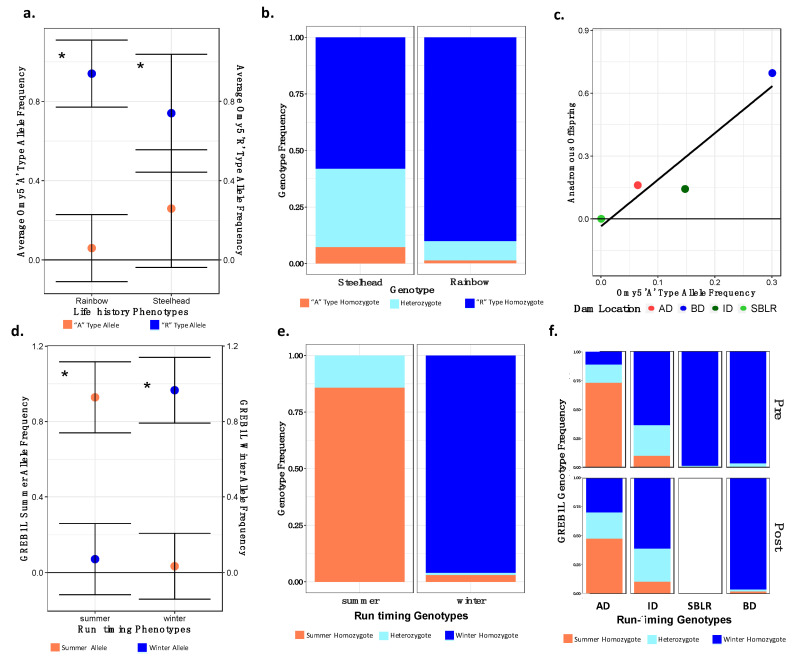
There were significant differences in the allelic and genotypic frequencies of the Omy5 (**a**–**c**) and the GREB1L variants (**d**–**f**) among populations and life histories. There were significant differences in the mean allele frequencies of the Omy5 locus allele frequencies between steelhead and unknown forms (**a**) and the frequency of the Omy5 “A” type allele frequency significantly explained life history phenotype (**b**). We also observed a strong correlation between the number of anadromous individuals from each population and the frequency of the “A” allele (**c**). The mean frequency of the GREB1L summer allele also varied significantly between summer- and winter-run steelhead (**d**), with the frequency of the homozygous summer genotype significantly explaining run timing (**e**), and inferred, ancestral population explaining a significant proportion of the variation in genotype both prior to and post-dam removal (**f**). Asterisks (**a**,**c**) indicate statistical significance (α = 0.01) (*).

## 3. Modified Main Text Tables

Original Table 1:

**Table 1:** All sequenced samples arranged by population (AD, ID, SBLR, and BD) and sampling site, ordered from upstream (top) to downstream (bottom) divided by relative anadromous barrier location.

Relative Dam Location Sampling Site	Prior to Dam Removal	Post-Dam Removal
**Above the Dams (AD)**	**208**	**0**
Cat Creek	21	
Chicago Camp	7	
Elkhorn	37	
Geyser	39	
Hayes	35	
Whiskey Bend	25	
Wilder	44	
**In between the Dams (ID)**	**169**	**297**
Aldwell	20	
Altaire	32	
Campground Creek	30	
Elwha River	0	143
Indian Creek	13	146
Little River	49	8
Madison Creek	25	
**South Branch of the Little River (SBLR)**	**86**	**0**
**Below the Dams (BD)**	**104**	**261**
**Total**	**567**	**558**

Modified Table 1: Updated table, accounting for updated tallies once metadata errors were corrected.

**Table 1:** All sequenced samples arranged by population (AD, ID, SBLR, and BD) and sampling site, ordered from upstream (top) to downstream (bottom) divided by relative anadromous barrier location.

Relative Dam Location Sampling Site	Prior to Dam Removal	Post-Dam Removal
**Above the Dams (AD)**	**208**	**3**
Cat Creek	21	
Chicago Camp	7	
Elkhorn	37	
Elwha River		3
Geyser	39	
Hayes	35	
Whiskey Bend	25	
Wilder	44	
**In between the Dams (ID)**	**169**	**304**
Aldwell	20	
Altaire	32	
Campground Creek	30	
Elwha River		150
Indian Creek	13	146
Little River	49	8
Madison Creek	25	
**South Branch of the Little River (SBLR)**	**86**	**0**
**Below the Dams (BD)**	**104**	**249**
**Total**	**567**	**556**

Original Table 3:

**Table 3:** The mean probability of assignment to each population represented in the steelhead life history cohorts sampled post-dam removal varied temporally. The below-dam population represented the largest proportion of genetic ancestry in the first steelhead life history cohorts recolonizing the Elwha River following dam removal with increased representation from above- and in-between-dam populations.

Steelhead Life History Cohort	Mean Posterior Probability				Mean Posterior Probability Credible Interval			
	AD	ID	SBLR	BD	AD	ID	SBLR	BD
**Adults Pre-2015**	0.096	0.078	0.001	0.825	0.036–0.180	0.024–0.159	0–0.014	0.723–0.910
**Adults 2015**	0.066	0.064	0.000	0.870	0.034–0.106	0.033–0.105	0–0.004	0.815–0.916
**Adults 2016**	0.058	0.099	0.003	0.840	0.004–9.179	0.018–0.237	0–0.038	0.673–0.948
**Adults 2017**	0.073	0.073	0.004	0.851	0.006–0.218	0.004–0.216	0–0.042	0.667–0.969
**Juveniles 2016**	0.198	0.223	0.000	0.578	0.140–0.257	0.164–0.287	0–0.004	0.502–0.654
**Juveniles 2017**	0.330	0.205	0.001	0.465	0.248–0.415	0.135–0.279	0–0.006	0.376–0.561

Modified Table 3: Numbers were updated following re-analysis after correction of individuals with metadata errors. 

**Table 3:** The mean probability of assignment to each population represented in the steelhead life history cohorts sampled post-dam removal varied temporally. The below-dam population represented the largest proportion of genetic ancestry in the first steelhead life history cohorts recolonizing the Elwha River following dam removal with increased representation from above- and in-between-dam populations.

Mixture Collection	Mean Posterior Probability				Mean Posterior Probability Credible Interval			
	AD	ID	SBLR	BD	AD	ID	SBLR	BD
**Adults Pre 2015**	0.094	0.0789	0.001	0.825	0.035–0.183	0.024–0.165	0–0.012	0.716–0.913
**Adults 2015**	0.067	0.065	0.000	0.868	0.034–0.109	0.032–0.105	0–0.005	0.813–0.916
**Adults 2016**	0.057	0.100	0.003	0.840	0.004–0.175	0.016–0.239	0–0.035	0.673–0.956
**Adults 2017**	0.080	0.074	0.003	0.842	0.006–0.246	0.005–0.223	0–0.035	0.644–0.969
**Juveniles 2016**	0.198	0.223	0.000	0.578	0.141–0.257	0.164–0.288	0–0.004	0.502–0.654
**Juveniles 2017**	0.330	0.206	0.001	0.464	0.243–0.414	0.138–0.282	0–0.006	0.374–0.562

## 4. Modified Appendix Figures

Original Figure A1:

**Figure A1:**
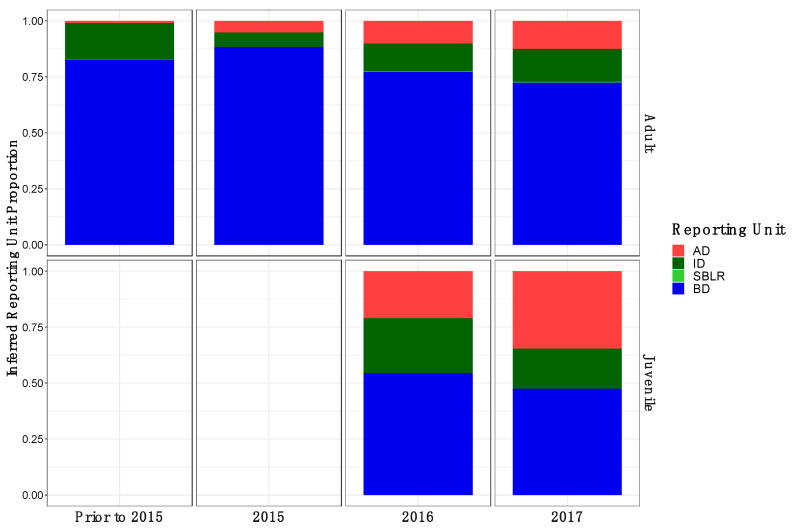
Inferred proportion of genetic ancestry from each of the four inferred populations across recolonizing adult and juvenile steelhead smolt life history cohorts from the GSI analysis (ii). Colors are representative of the inferred population. The bottom x axis divides life history cohorts temporally and the right y axis divides them by life history stage. Relative proportions of genetic ancestry from ID populations were greater in adult life history cohorts in GSI analysis (ii) compared to GSI analysis (i) and GSI analysis (iii).

Modified Figure A1: Updated following correction to metadata.

**Figure A1:**
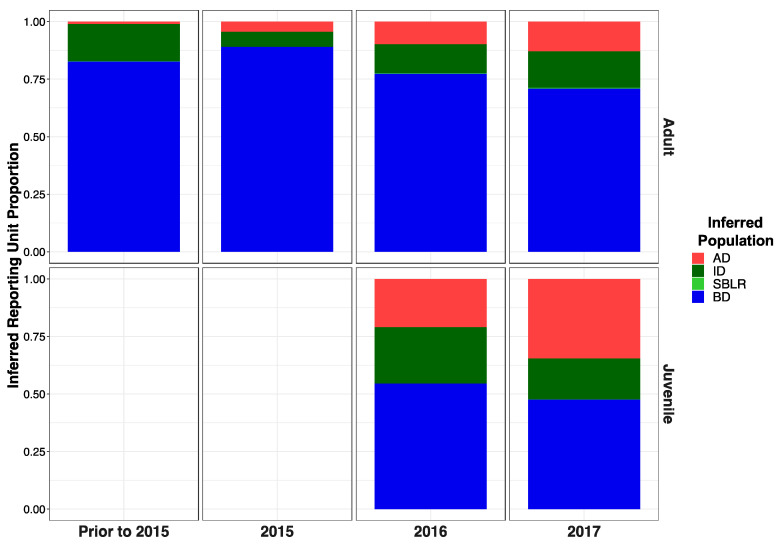
Inferred proportion of genetic ancestry from each of the four inferred populations across recolonizing adult and juvenile steelhead smolt life history cohorts from the GSI analysis (ii). Colors are representative of the inferred population. The bottom x axis divides life history cohorts temporally and the right y axis divides them by life history stage. Relative proportions of genetic ancestry from ID populations were greater in adult life history cohorts in GSI analysis (ii) compared to GSI analysis (i) and GSI analysis (iii).

Original Figure A2:

**Figure A2:**
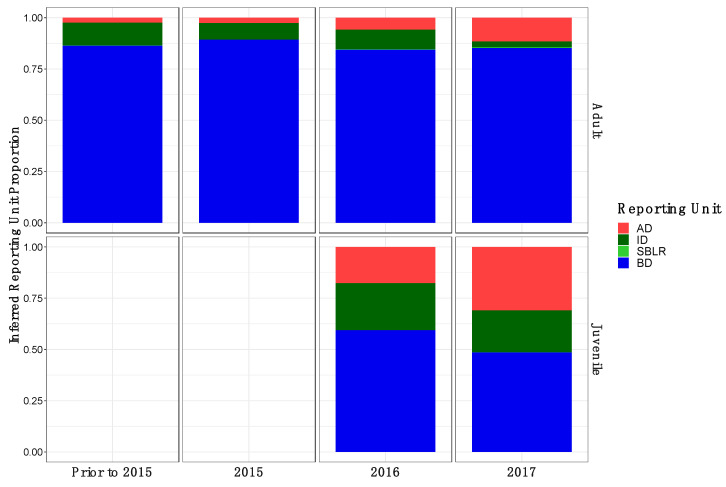
Inferred proportion of genetic ancestry from each of the four inferred populations across recolonizing adult and juvenile steelhead smolt life history cohorts from the GSI analysis (iii). Relative proportions of genetic ancestry from BD populations were greater in adult life history cohorts in GSI analysis (iii) compared to GSI analysis (i) GSI analysis (ii).

Modified Figure A2: Modified following correction to metadata.

**Figure A2:**
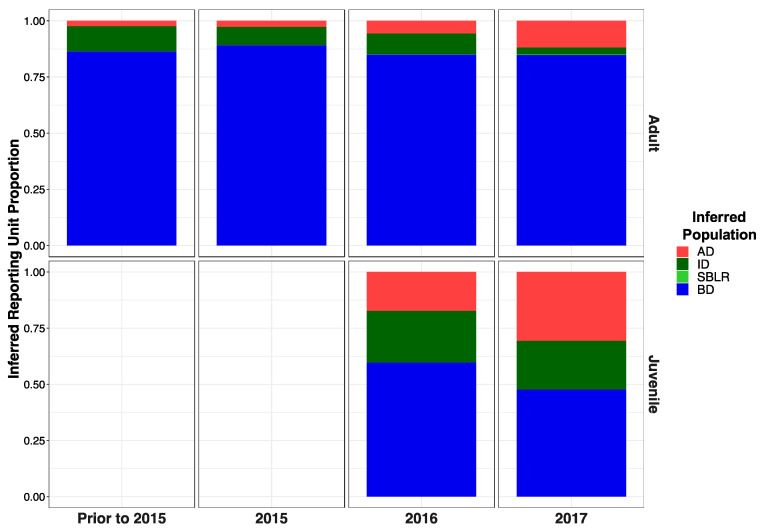
Inferred proportion of genetic ancestry from each of the four inferred populations across recolonizing adult and juvenile steelhead smolt life history cohorts from the GSI analysis (iii). Relative proportions of genetic ancestry from BD populations were greater in adult life history cohorts in GSI analysis (iii) compared to GSI analysis (i) GSI analysis (ii).

The authors apologize for any inconvenience caused and state that the scientific conclusions are unaffected. The original publication and the supplementary materials has also been updated.
