# Supplementary material for: The Impacts of Dam Construction and Removal on the Genetics of Recovering Steelhead (Oncorhynchus mykiss) Populations across the Elwha River Watershed
Source: Genes (Basel). 2021 Jan 13;12(1):89. doi: 10.3390/genes12010089 (PMC7828262; doi:10.3390/genes12010089)
Supplement: Supplementary file 1 [file genes-12-00089-s001.pdf]

Back matter: Supplementary Materials, Acknowledgments, Author Contributions, Conflicts of Interest, References.

Supplementary Materials

Tables

	Population	Sample Size	Sum of Quality Filtered Reads	Sum of Clone Filtered Read Pairs	Sum of Mapped Read Pairs	Average Percent Reads Mapped
**Pre-Dam Removal**	Above the Dams (AD)	208	214853211	74787568	146324196	97.48
	In Between the Dams (ID)	169	194678581	70081716	137204353	97.46
	South Branch of the Little River (SBLR)	86	89071386	34666100	67962209	97.39
	Below the Dams (BD)	104	128606386	37176209	72791991	97.41
**Post-Dam Removal**	In Between the Dams (ID)	288	439358042	146791213	287173372	97.16
	Below the Dams (BD)	270	649232832	177443163	346936489	97.32

Supplementary Table 1: Summary alignment and quality filtering statistics for read pairs across the 1,125 samples retained for downstream analyses from the original RADsequencing of 1,334 samples.

Supplementary Table 2: Mean population genetics statistics calculated in VCFtools (π, Tajima's D, A_{jk}), N_eEstimator v2 (N_e), and the inbreeding coefficient (F_{IS}) temporally, across sampling location, among life history forms, and across life history cohorts. Confidence intervals represent non-parametric bootstrapped 95% confidence intervals for all estimates except for N_e which represent internally calculated 95% credible intervals.

		Nucleotide Diversity (π)	Tajima's D	Relatedness (A_{jk})	Inbreeding Coefficient (F_{IS})	Effective Population Size (N_e)	Total non-polymorphic loci	Number of Individuals
**Prior to dam removal**	**All**	0.056 ± 0.078	-0.42 ± 0.58	0.0017 ± 0.072	0.030 ± 0.10	80.3.1 (66.6-97.0)	1,230	567
	**Unknown**	0.056 ± 0.080	-0.41 ± 0.518	0.0021 ± 0.081	0.032 ± 0.10	58.4 (49.7-68.5)	1,617	463
	**Steelhead**	0.053 ± 0.081	-0.60 ± 0.553	0.0090 ± 0.140	-0.0060 + 0.10	141.3 (102.8-213.8)	7,152	104
	**AD**	0.053 ± 0.083	-0.46 ± 0.578	0.0047 ± 0.11	0.027 ± 0.11	120.9 (97.1-154.6)	7,563	208
	**ID**	0.061 ± 0.085	-0.449 ± 0.562	0.0057 ± 0.11	0.028 ± 0.11	150.7 (109.4-114.7)	4,127	169
	**SBLR**	0.044 ± 0.11	-0.082 ± 0.899	0.018 ± 0.16	0.011 ± 0.12	38.3 (26.5-58.3)	28,727	86
	**BD**	0.053 ± 0.081	-0.60 ± 0.556	0.0090 ± 0.14	-0.038 ± 0.16	141.3 (102.8-213.8)	7,144	104
**Post dam removal**	**All (Steelhead)**	0.058 ± 0.083	-0.36 ± 0.55	0.0018 ± 0.066	0.011 ± 0.078	140.2 (117.5-169.5)	3,786	558
	**Adults Pre 2015**	0.054 ± 0.086	-0.624 ± 0.59	0.017 ± 0.19	0.0079 ± 0.11	159.6 (99.3-356.4)	10,159	57
	**Adults 2015**	0.057 ± 0.09	-0.437 ± 0.62	0.0047 ± 0.11	0.009 ± 0.10	63.9 (53.1-77.6)	5,859	173
	**Adults 2016**	0.060 ± 0.10	-0.645 ± 0.65	0.0047 ± 0.11	0.0065 ± 0.17	132.1 (80.6-332.1)	16,008	24
	**Adults 2017**	0.060 ± 0.10	-0.656 ± 0.668	0.049 ± 0.32	0.011 ± 0.18	22.4 (7.7-INF)	17,921	19
	**Juveniles 2016**	0.058 ± 0.082	-0.511 ± 0.552	0.0059 ± 0.11	0.0067 ± 0.10	264.9 (194.3-311.8)	2,176	166
	**Juveniles 2017**	0.057 ± 0.083	-0.56 ± 0.554	0.0081 ± 0.13	0.003 ± 0.12	341.2 (323.2-347.4)	3,570	119

**Sampling Site**	**Inferred Sampling Site**													
	Chicago Camp	Wilder	Hayes	Elkhorn	Geyser	Cat Creek	Altaire	Campground Creek	Madison Creek	Little River	Indian Creek	Aldwell	South Branch Little River	Elwha River
Chicago Camp	**1.000**	0.000	0.000	0.000	0.000	0.000	0.000	0.000	0.000	0.000	0.000	0.000	0.000	0.000
Wilder	0.000	**0.246**	0.156	0.442	0.043	0.114	0.000	0.000	0.000	0.000	0.000	0.000	0.000	0.000
Hayes	0.000	0.272	**0.215**	0.384	0.000	0.129	0.000	0.000	0.000	0.000	0.000	0.000	0.000	0.000
Elkhorn	0.000	0.032	0.078	**0.823**	0.066	0.001	0.000	0.000	0.000	0.000	0.000	0.000	0.000	0.000
Geyser	0.000	0.027	0.007	0.319	**0.542**	0.105	0.000	0.000	0.000	0.000	0.000	0.000	0.000	0.000
Cat Creek	0.000	0.000	0.000	0.015	0.985	**0.000**	0.000	0.000	0.000	0.000	0.000	0.000	0.000	0.000
Altaire	0.000	0.000	0.000	0.000	0.000	0.004	**0.314**	0.341	0.074	0.132	0.000	0.136	0.000	0.000
Campground Creek	0.000	0.000	0.000	0.000	0.000	0.105	0.239	**0.233**	0.102	0.193	0.000	0.128	0.000	0.000
Madison Creek	0.000	0.000	0.000	0.000	0.000	0.000	0.046	0.033	**0.581**	0.259	0.000	0.081	0.000	0.000
Little River	0.000	0.000	0.000	0.000	0.000	0.000	0.000	0.000	0.060	**0.917**	0.000	0.023	0.000	0.000
Indian Creek	0.000	0.000	0.000	0.000	0.000	0.000	0.000	0.000	0.000	0.000	**1.000**	0.000	0.000	0.000
Aldwell	0.000	0.000	0.000	0.000	0.000	0.000	0.255	0.274	0.125	0.250	0.000	**0.096**	0.000	0.000
South Branch Little River	0.000	0.000	0.000	0.000	0.000	0.012	0.000	0.000	0.000	0.000	0.000	0.000	**0.976**	0.012
Elwha River	0.000	0.000	0.000	0.000	0.000	0.148	0.006	0.001	0.011	0.001	0.000	0.000	0.000	**0.833**

Supplementary Table 3: The results from simulations performed on all 479 individuals included in the reference collections reflected mixed results for accuracy of assignment of individual fish back to their respected reference collections, or sampling sites, based on their genotypes. The probabilities for self-assignment of individuals back to their sampling site were calculated by taking the mean of the scaled likelihoods. The scaled likelihoods reflect the probability of assigning an individual fish to the inferred collection.

Life history Cohort	Sample ID	Inferred Population	Inferred Sampling Site	Posterior Probability of Membership	Log Likelihood
Prior to Dam Removal	34188_008	ID	Altaire	0.553	-191.159
Prior to Dam Removal	33649_39	ID	Aldwell	0.650	-231.978
Prior to Dam Removal	34118_053	ID	Aldwell	0.898	-296.727
Adults Sampled Pre 2015	51029_E13_004b	AD	Cat Creek	0.720	-247.024
Juveniles 2016	51659_M_042816_smo_2	AD	Elkhorn	0.886	-234.457
Juveniles 2017	51786_M_050217_28	AD	Cat Creek	0.882	-233.127

Supplementary Table 4: Inferred reporting units for the six individuals that had low (< 0.9) posterior probabilities of membership to their inferred collection.

Sampling Sites	Sample ID	Above the Dams (AD)						In Between the Dams (ID)						South Branch Little River (SBLR)	Below the Dams (BD)
		Chicago Camp	Wilder	Hayes	Elkhorn	Geyser	Cat Creek	Altaire	Campground Creek	Madison Creek	Little River	Indian Creek	Aldwell		
Elkhorn	33696_011	0	0	0.01	0	0.988	0.002	0	0	0	0	0	0	0	0
Geyser	34186_032	0	0	0	0	0	0	0	0	0	1	0	0	0	0
Geyser	34186_041	0	0	0	0	0	0	0	0	0	1	0	0	0	0
Whiskey Bend	34185_001	0	0	0	0	0	0	0	0	0	1	0	0	0	0
Whiskey Bend	34185_002	0	0	0	0	0	0	0	0	0	1	0	0	0	0
Whiskey Bend	34185_003	0	0	0	0	0	0	0	0	0	0	0	0	0	1
Whiskey Bend	34185_004	0	0	0	0	0	0	0	0	0	0	0	0	0	1
Whiskey Bend	34185_005	0	0	0	0	0	0	0	0	0	1	0	0	0	0
Whiskey Bend	34185_006	0	0	0	0	0	0	0.219	0.781	0	0	0	0	0	0
Whiskey Bend	34185_007	0	0	0	0	0	0	0.775	0.004	0.214	0.006	0	0	0	0
Whiskey Bend	34185_008	0	0	0	0	0	0	1	0	0	0	0	0	0	0
Whiskey Bend	34185_009	0	0	0	0	0	0	0	0	0	1	0	0	0	0
Whiskey Bend	34185_010	0	0	0	0	0	0	1	0	0	0	0	0	0	0
Whiskey Bend	34185_011	0	0	0	0	0	0	0	0	0	1	0	0	0	0
Whiskey Bend	34185_012	0	0	0	0	0	0	0.801	0.003	0.195	0	0	0.001	0	0
Whiskey Bend	34185_013	0	0	0	0	0	0	0	1	0	0	0	0	0	0
Whiskey Bend	34185_014	0	0	0	0	0	0	0	0	0	1	0	0	0	0
Whiskey Bend	34185_015	0	0	0	0	0	0	0.001	0	0	0.999	0	0	0	0
Whiskey Bend	34185_016	0	0	0	0	0	0	0	0	0	1	0	0	0	0
Whiskey Bend	34185_017	0	0	0	0	0	0	0.149	0.003	0.849	0	0	0	0	0
Whiskey Bend	34185_018	0	0	0	0	0	0	0	0	0	1	0	0	0	0
Whiskey Bend	34185_019	0	0	0	0	0	0	1	0	0	0	0	0	0	0
Whiskey Bend	34185_020	0	0	0	0	0	0	0	0	0	1	0	0	0	0
Whiskey Bend	34185_021	0	0	0	0	0	0	0	0	0	0	0	0	0	1

Sampling Sites	Sample ID	Above the Dams (AD)						In Between the Dams (ID)						South Branch Little River (SBLR)	Below the Dams (BD)
		Chicago Camp	Wilder	Hayes	Elkhorn	Geyser	Cat Creek	Altaire	Campground Creek	Madison Creek	Little River	Indian Creek	Aldwell		
Whiskey Bend	34185_022	0	0	0	0	0	0	0.026	0	0	0.974	0	0	0	0
Whiskey Bend	34185_023	0	0	0	0	0	0	0	0	0	1	0	0	0	0
Whiskey Bend	34185_024	0	0	0	0	0	0	0.002	0	0	0.997	0	0	0	0
Whiskey Bend	34185_025	0	0	0	0	0	0	0	0	0	1	0	0	0	0
Cat Creek	34188_001	0	0	0	0	0	0	0	0	0	1	0	0	0	0
Cat Creek	34188_003	0	0	0	0	0	0.948	0	0	0	0.052	0	0	0	0
Cat Creek	34188_004	0	0	0	0	0	0	0	1	0	0	0	0	0	0
Cat Creek	34188_005	0	0	0	0.781	0	0	0	0	0	0	0	0	0	0.22
Cat Creek	34188_006	0	0	0	0	0	0	0	0	0	1	0	0	0	0
Cat Creek	34188_007	0	0	0	0	0	0	0	0	1	0	0	0	0	0
Cat Creek	34188_009	0	0	0	0	0	0	0.073	0.927	0	0	0	0	0	0
Cat Creek	34188_010	0	0	0	0	0	0	0	0	0	1	0	0	0	0
Cat Creek	34188_012	0	0	0	0	0	0	1	0	0	0	0	0	0	0
Cat Creek	34188_013	0	0	0	0	0	0	0.999	0	0	0.001	0	0	0	0
Cat Creek	34188_014	0	0	0	0	0	1	0	0	0	0	0	0	0	0
Cat Creek	34188_015	0	0	0	0	0	1	0	0	0	0	0	0	0	0
Cat Creek	34188_016	0	0	0	0	0	0	0.001	0.991	0	0	0	0.008	0	0
Cat Creek	34188_019	0	0	0	0	0	0	0	0	0.002	0.998	0	0	0	0
Cat Creek	34188_021	0	0	0	0	0	0	1	0	0	0	0	0	0	0
Cat Creek	34188_022	0	0	0	0	0	0	0	0	0.129	0.871	0	0	0	0
Cat Creek	34188_011	0	0	0	0	0	0.932	0.067	0	0	0	0	0	0	0
Altaire	33694_027	0	0	0	0	0	0	0	0.027	0.965	0.004	0	0.005	0	0
Altaire	33694_058	0	0	0	0	0	0	0.995	0.003	0	0.002	0	0	0	0
Altaire	33694_095	0	0	0	0	0	0	1	0	0	0	0	0	0	0

Sampling Sites	Sample ID	Above the Dams (AD)						In Between the Dams (ID)						South Branch Little River (SBLR)	Below the Dams (BD)
		Chicago Camp	Wilder	Hayes	Elkhorn	Geyser	Cat Creek	Altaire	Campground Creek	Madison Creek	Little River	Indian Creek	Aldwell		
Altaire	33694_097	0	0	0	0	0	0	1	0	0	0	0	0	0	0
Altaire	34189_003	0	0	0	0	0	0	0	1	0	0	0	0	0	0
Campground Creek	34190_001	0	0	0	0	0	0	0	0	0	1	0	0	0	0
Campground Creek	34190_005	0	0	0	0	0	0	0	0	0	1	0	0	0	0
Campground Creek	34190_008	0	0	0	0	0	1	0	0	0	0	0	0	0	0
Campground Creek	34190_012	0	0	0	0	0	0	0	0	0	1	0	0	0	0
Campground Creek	34190_017	0	0	0	0	0	0	0.002	0.998	0	0	0	0	0	0
Campground Creek	34190_019	0	0	0	0	0	0	0.965	0.035	0	0	0	0	0	0
Campground Creek	34190_020	0	0	0	0	0	0	0	1	0	0	0	0	0	0
Campground Creek	34190_024	0	0	0	0	0	0	1	0	0	0	0	0	0	0
Campground Creek	34190_025	0	0	0	0	0	0	0.947	0.053	0	0	0	0	0	0
Campground Creek	34190_031	0	0	0	0	0	0	0.992	0	0	0	0	0.008	0	0
Campground Creek	34190_034	0	0	0	0	0	0	0.969	0	0.025	0.006	0	0	0	0
Little River	33649_31	0	0	0	0	0	0	0	0	0	1	0	0	0	0
Little River	33649_53	0	0	0	0	0	0	0	0	0	1	0	0	0	0
Little River	33649_55	0	0	0	0	0	0	0	0	0	1	0	0	0	0
Indian Creek	33675_246	0	0	0	0	0	0	0	0	0	0	1	0	0	0
Indian Creek	33675_256	0	0	0	0	0	0	0	0	0	0	1	0	0	0
Indian Creek	33675_258	0	0	0	0	0	0	0	0	0	0	1	0	0	0
Aldwell	34118_041	0	0	0	1	0	0	0	0	0	0	0	0	0	0

Sampling Sites	Sample ID	Above the Dams (AD)						In Between the Dams (ID)						South Branch Little River (SBLR)	Below the Dams (BD)
		Chicago Camp	Wilder	Hayes	Elkhorn	Geyser	Cat Creek	Altaire	Campground Creek	Madison Creek	Little River	Indian Creek	Aldwell		
Aldwell	34118_049	0	0	0	0	0	0	0.048	0.95	0.001	0	0	0	0	0
Aldwell	34118_151	0	0	0	1	0	0	0	0	0	0	0	0	0	0
Aldwell	34118_152	0	0	0	0	0	1	0	0	0	0	0	0	0	0
Aldwell	34118_153	0	0	0	0	0	1	0	0	0	0	0	0	0	0
Aldwell	34118_154	0	0	0	1	0	0	0	0	0	0	0	0	0	0
Aldwell	34118_155	0	0.001	0	0.999	0	0	0	0	0	0	0	0	0	0
Aldwell	34118_156	0	0	0	1	0	0	0	0	0	0	0	0	0	0
Aldwell	34118_169	0	0	0	0	0	0	1	0	0	0	0	0	0	0
Aldwell	34118_171	0	0	0	0	0	0	1	0	0	0	0	0	0	0
Aldwell	34118_174	0	0	0	0	0	0	0	0	1	0	0	0	0	0
SBLR	33651_125	0	0	0	0	0	1	0	0	0	0	0	0	0	0
SBLR	33651_140	0	0	0	0	0	1	0	0	0	0	0	0	0	0
SBLR	33830_25	0	0	0	0	0	1	0	0	0	0	0	0	0	0
Elwha River	34336_C5_012	0	0	0	0	0	0	0	0	0	0.99	0	0	0	0.001
Elwha River	34336_C5_018	0	0	0	0	0	0	0	0	0	0	0	0	0	1
Elwha River	51254_C2_029	0	0	0	0	0	0	0	0	0	0	0	0	0	1

Supplementary Table 5: The mean posterior probability of membership to reference collections for the 85 individuals not included in the reference sampling set due to incongruent sampling location and DAPC population assignments.

3 **Figures**

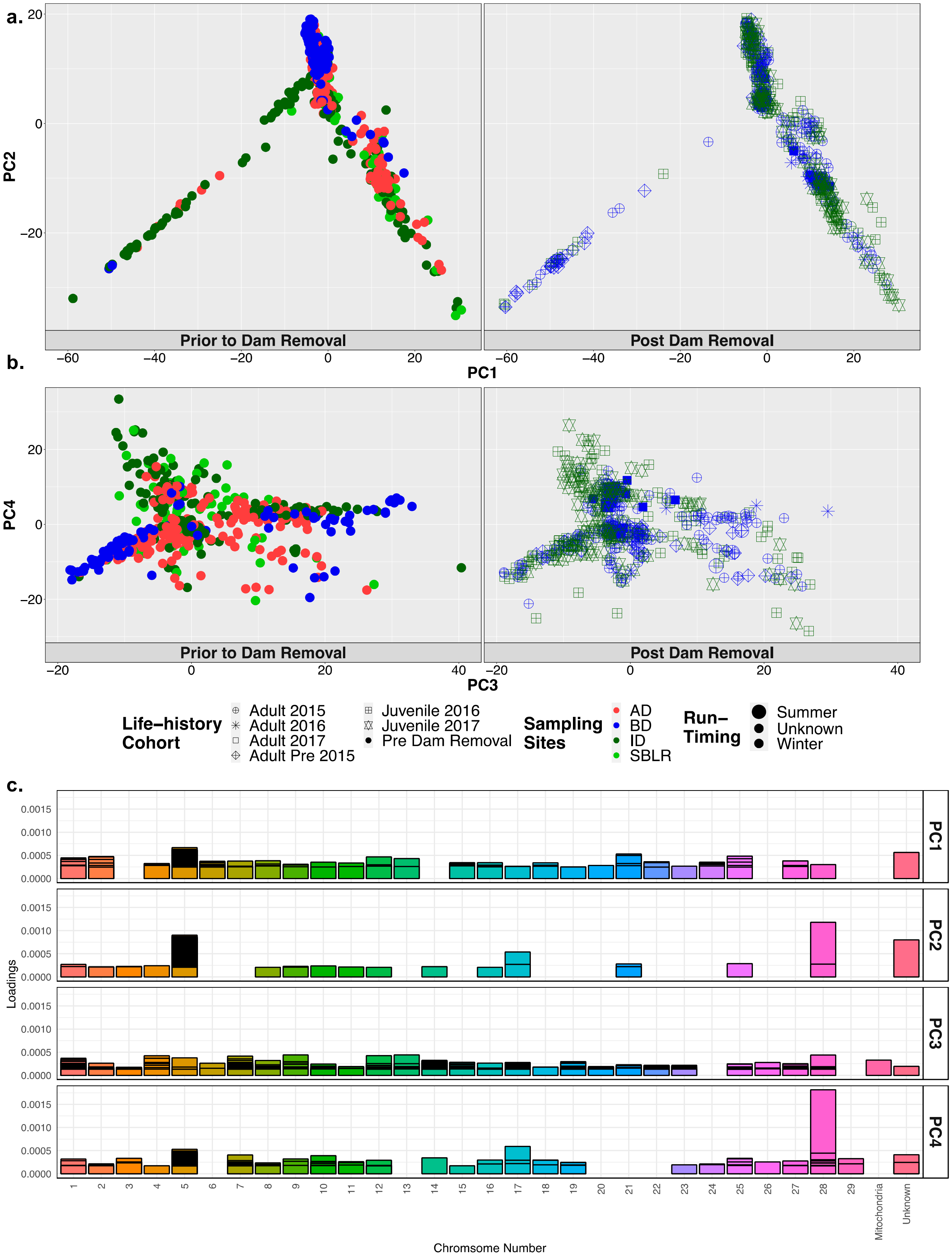
a.
b.
c.
PC1
PC2
PC3
PC4
Prior to Dam Removal
Post Dam Removal
Life−history Cohort
Adult 2015
Adult 2016
Adult 2017
Adult Pre 2015
Juvenile 2016
Juvenile 2017
Pre Dam Removal
Sampling Sites
AD
BD
ID
SBLR
Run−Timing
Summer
Unknown
Winter
Loadings
Chromsome Number
Mitochondria
Unknown

<u>Supplementary Figure 1</u>: There were no discernible clustering patterns in the PCA conducted among all 71,320 SNPs used in this study among the samples collected prior to or following dam removal among principal components one (proportion of variance explained = 2.87%) and two (2.12%) (a), or three (1.22%) and four (0.83%) (b). Points were colored based on the sampling site location relative to anadromous barrier. Point shapes were reflective of life history cohort and point size was indicative of known migratory life history phenotype. A large number of loci on Chromosome 5 (Omy5) loaded on the first, second, and fourth principal components and a small number of loci on Chromosome 28 (Omy28) with large loadings loaded on the second and fourth principal components (c). Each bar includes the top 1% SNPs across chromosomes loading on individual PCs. Black boxes within each bar represent a single SNP and the width of the black box is proportional to the cumulative proportion of the loadings explained by each SNP.

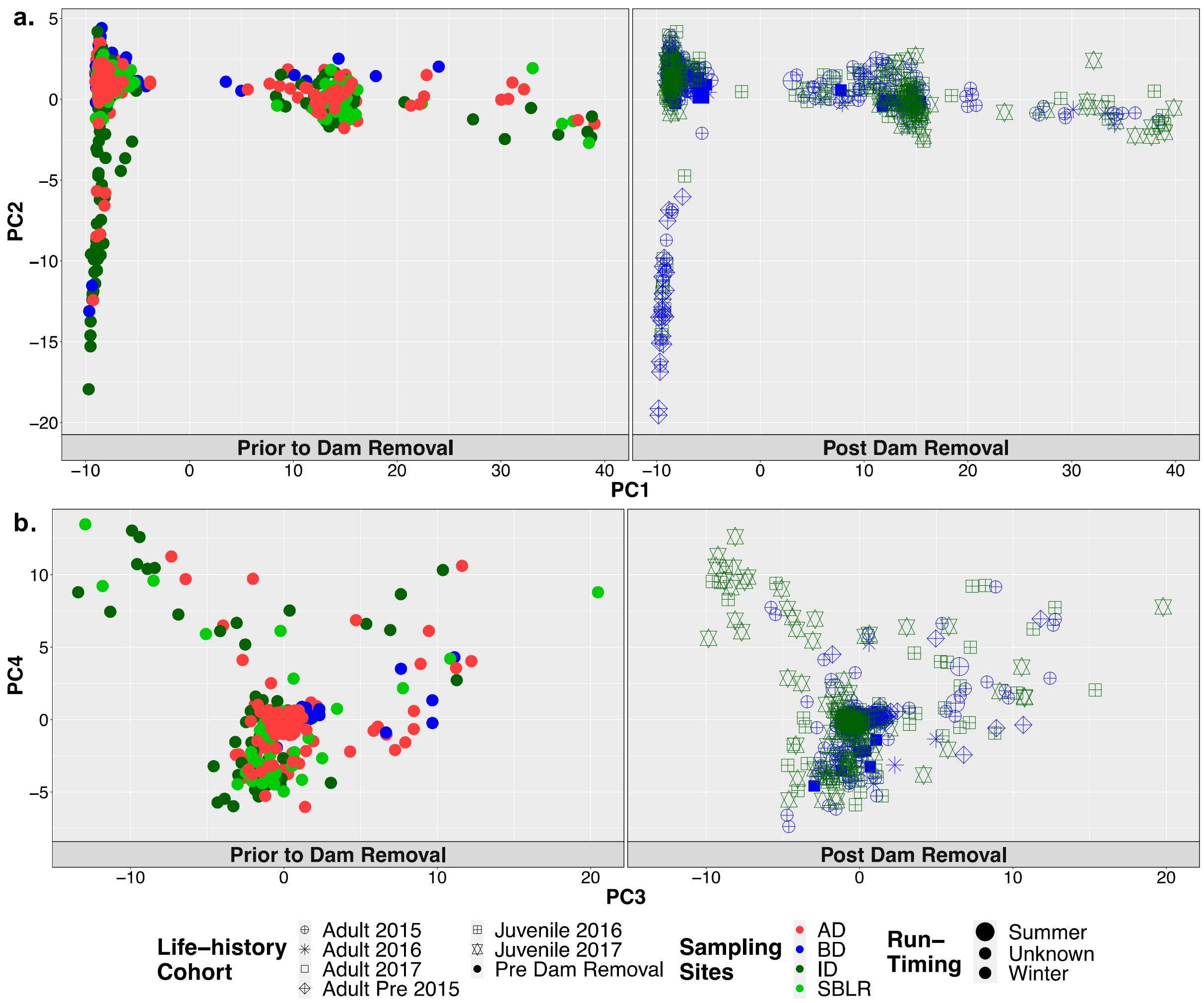

<u>Supplementary Figure 2</u>: There were three distinct clusters in the PCA conducted among all 2,552 SNPs called on Omy5 among the samples collected prior to or following dam removal among principal components one (proportion of variance explained = 27.16%) and two (2.08%) (a), or three (1.59%) and four (1.28%) (b). Points were colored based on the sampling site location relative to anadromous barrier. Point shapes were reflective of life history cohort and point size was indicative of known migratory life history phenotype.

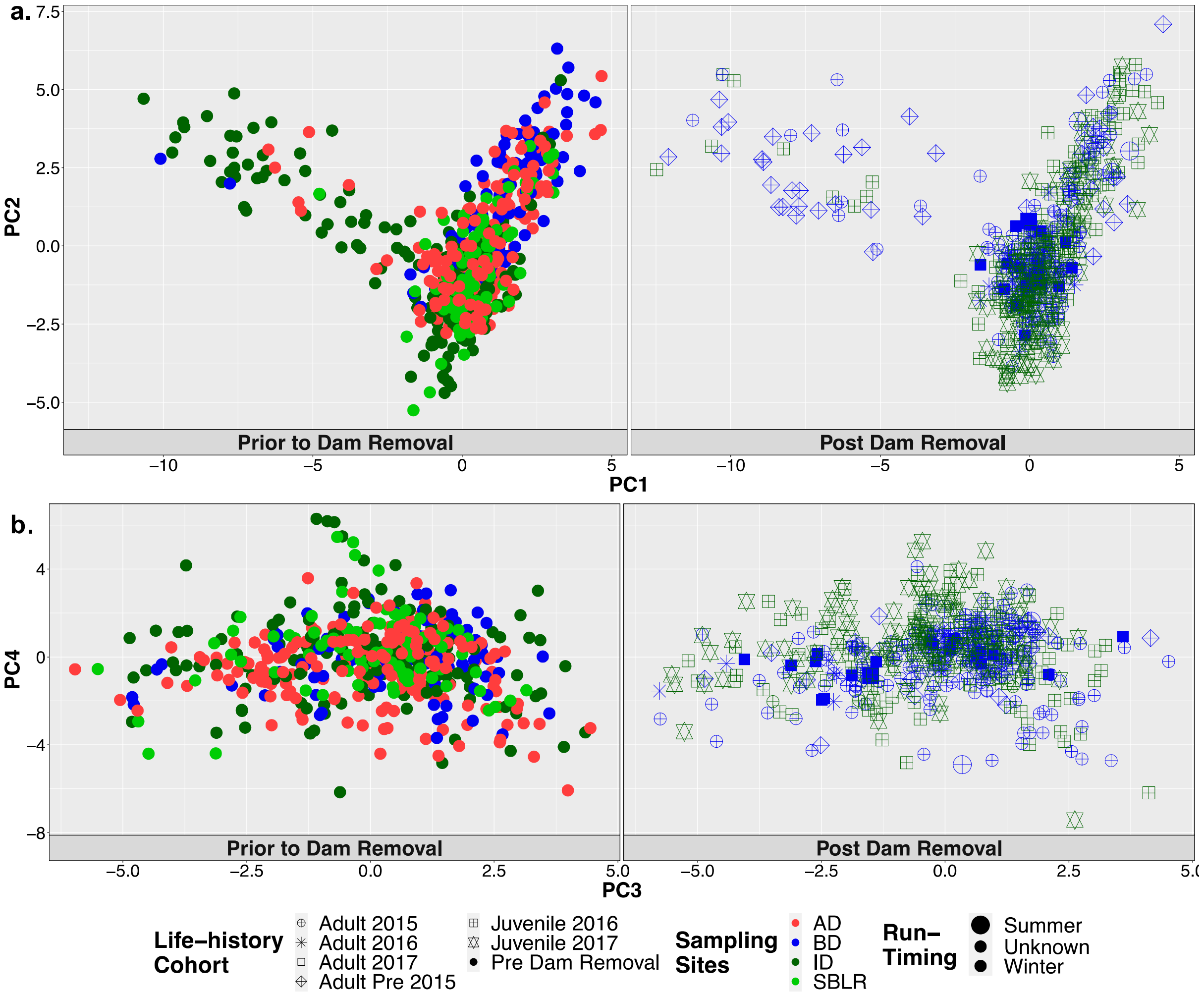

<u>Supplementary Figure 3</u>: There were no discernible clustering patterns in the PCA conducted among the 202 SNPs called on Omy28 among the samples collected prior to or following dam removal among principal components one (proportion of variance explained = 3.18%) and two (2.37%) (a), or three (1.63%) and four (1.46%) (b). Points were colored based on the sampling site location relative to anadromous barrier. Point shapes were reflective of life history cohort and point size was indicative of known migratory life history phenotype.

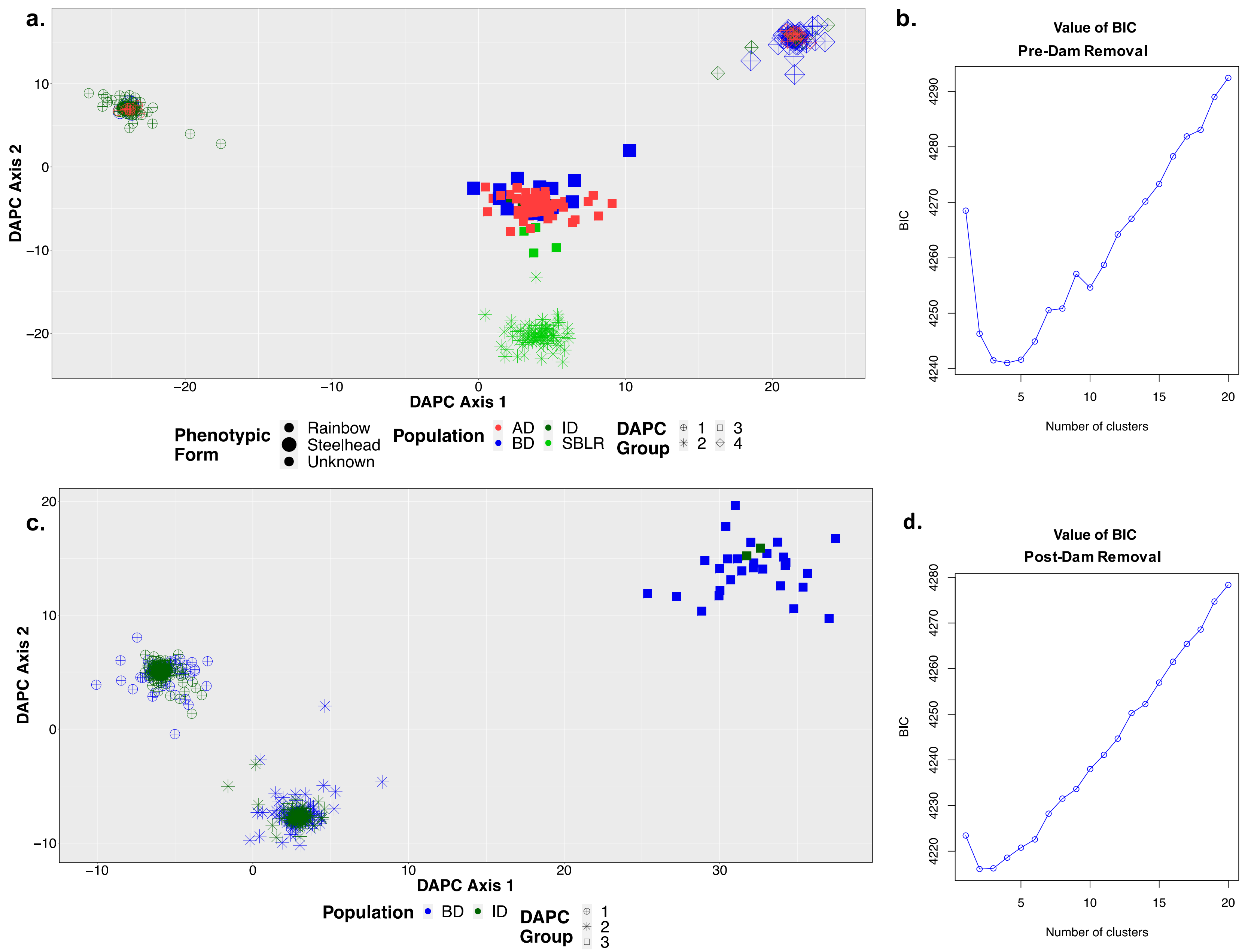
a.
b.
c.
d.
DAPC Axis 1
DAPC Axis 2
Phenotypic Form
Rainbow
Steelhead
Unknown
Population
AD
BD
ID
SBLR
DAPC Group
1
2
3
4
Value of BIC
Pre-Dam Removal
Post-Dam Removal
BIC
Number of clusters

Supplementary Figure 4: (a) DAPC results for the second-best K value (K=4) prior to dam removal and the (b) BIC plot. (c) The second-best supported structure model in DAPC post-dam removal was K =3 genetic clusters, (d) as shown by the elbow in the BIC plot. Shapes were indicative of population assignment by DAPC and color was based on sampling site location relative to the dams. Larger points in (a, b) were indicative of the Steelhead life history phenotypic form while smaller points were indicative of the resident Rainbow Trout form or fish that could not be categorized in either form. The elbow for BIC plots generated in DAPC genetic structure analysis of RAD-Sequenced samples supported (a) K=3 genetic clusters pre-dam removal (b) and K = 2 genetic clusters post-dam removal shown in Figure 4.

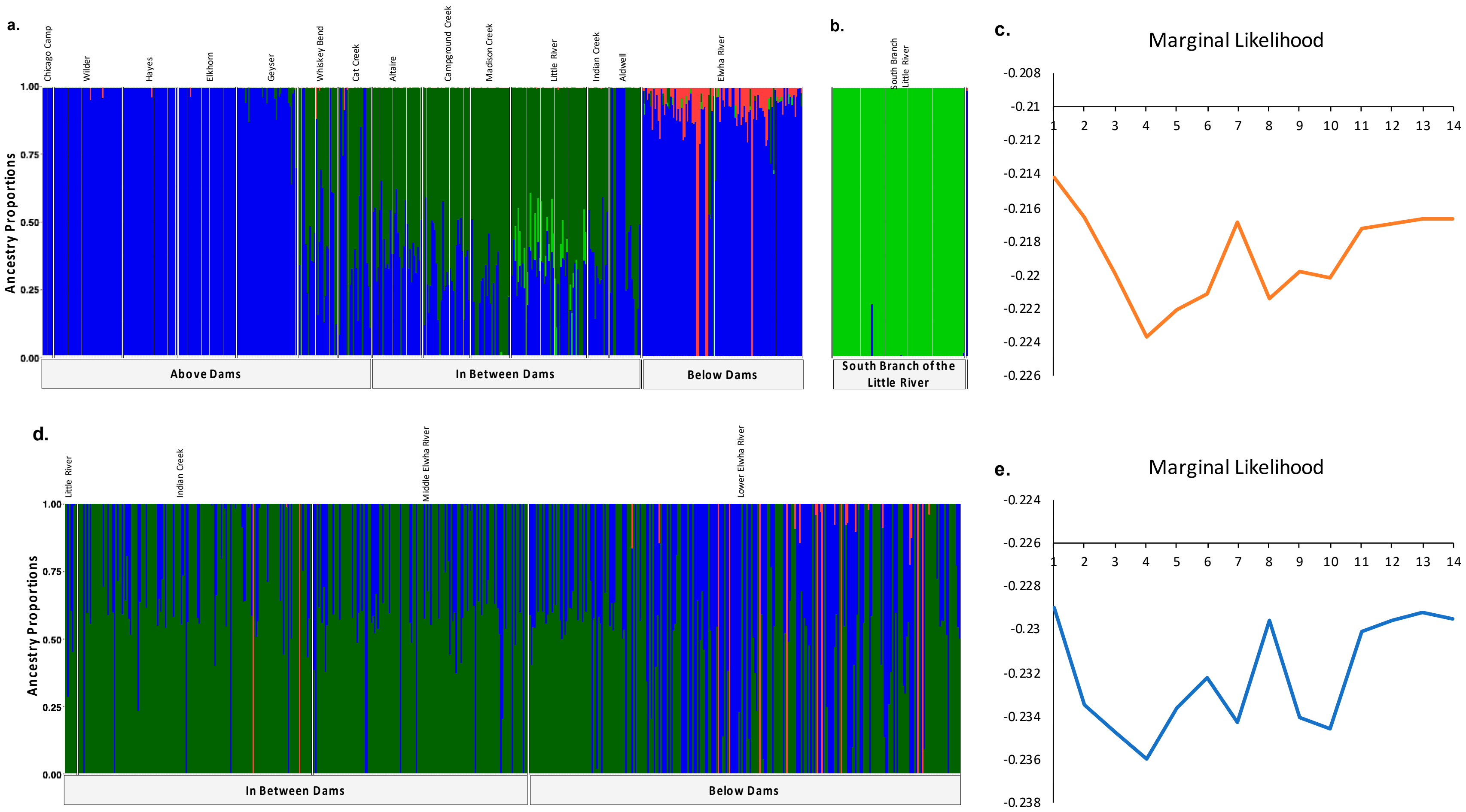

Supplementary Figure 5: FastSTRUCTURE population assignments for samples collected (a-b) prior to dam removal and supported four distinct genetic clusters (K=4). (a) High levels of genetic structure were observed among sampling locations split by dams (b) and natural barriers including South Branch Falls. (c) Marginal likelihood values were lowest at K=4 prior to dam removal, but model

components that explained structure support a K-value of 3 (Figure 4). (c) Post dam removal, we detected three genetic clusters respectively. (e) Marginal likelihood values were lowest at K=3 post-dam removal, but model components that explained structure support K=2 (Figure 4). Each vertical bar represents on fastSTRUCTURE plots (a-b,d) represented a single individual sampled at one of the sampling sites labelled across the top x-axis which are organized from up-river to down river and divided by anadromous barrier location which are labelled on the bottom x-axis. Each color represents a distinct genetic cluster.

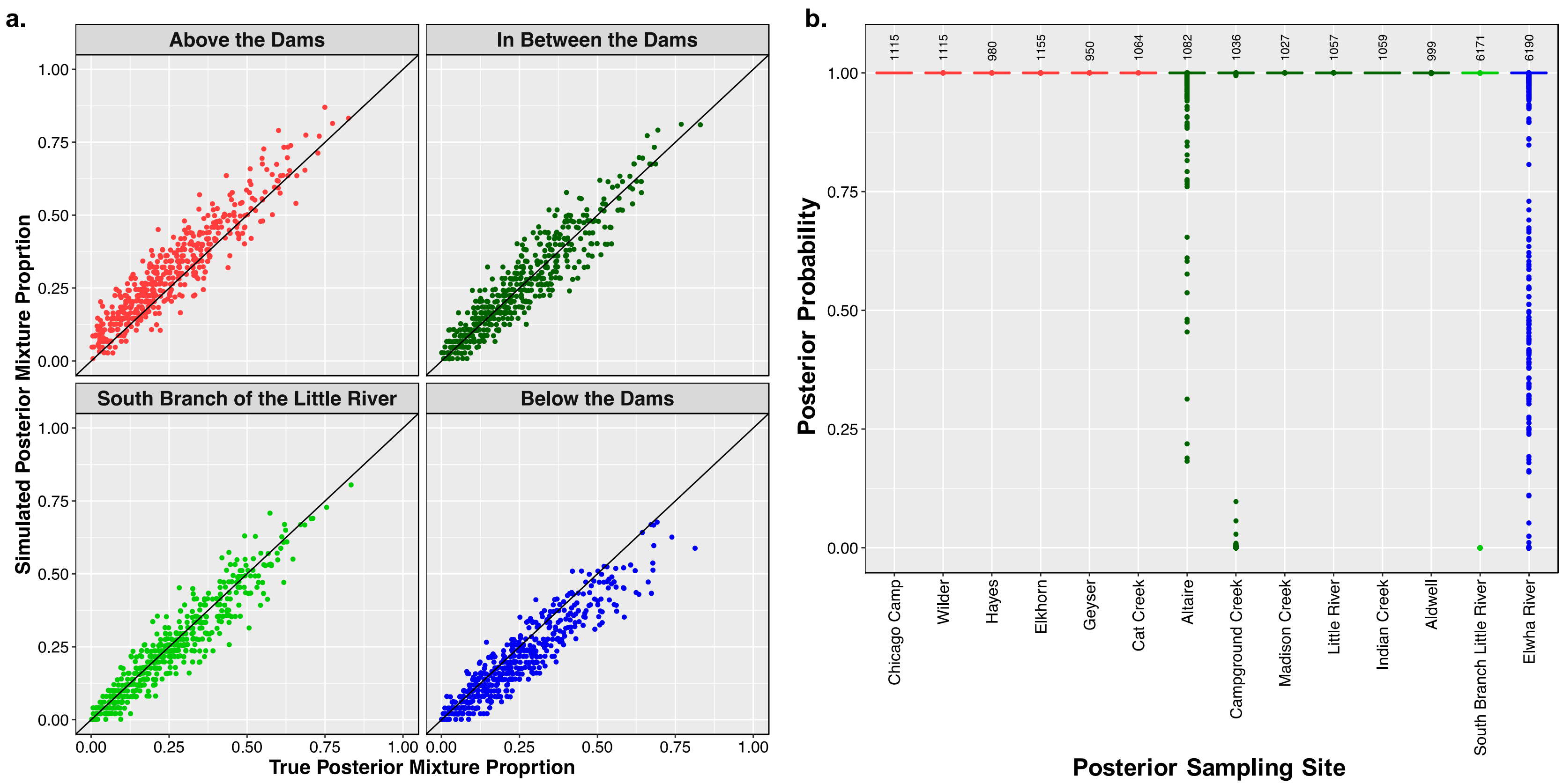

<u>Supplementary Figure 6</u>: (a) There was high correlation between the mixing proportions of genotypes sampled in simulated individuals to the true mixture proportions of the reference reporting units. This showed high accuracy of individual assignment to reporting units across all reference reporting units. (b) Simulated individuals representing the mixing proportions of the genotypes

sampled in the reference collections showed some variation in accuracy of individual assignment to reporting units across reference collections, particularly in the Elwha River, Campground Creek, Altaire, and South Branch of the Little River collections.

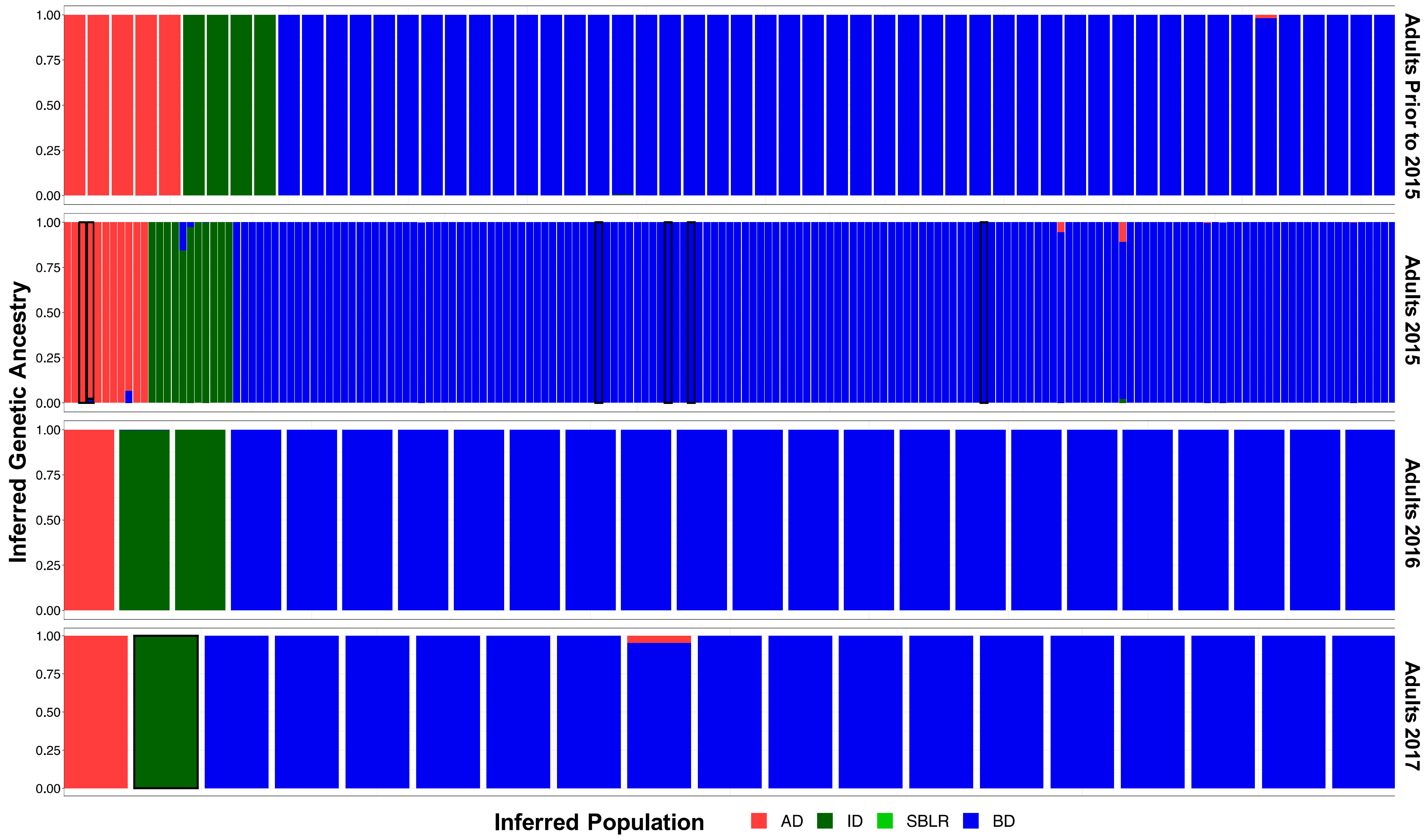

<u>Supplementary Figure 7</u>: The majority of GSI assignments computed by RUBIAS for adult Steelhead collected post dam removal were to BD reference collections and no individuals were assigned back to the SBLR. Each vertical bar represents a single individual

from one adult cohort labelled along the right y-axis. Colors represented the reference reporting unit or sampling location on the Elwha River. Adult individuals that were known to be summer Steelhead have a black outline.

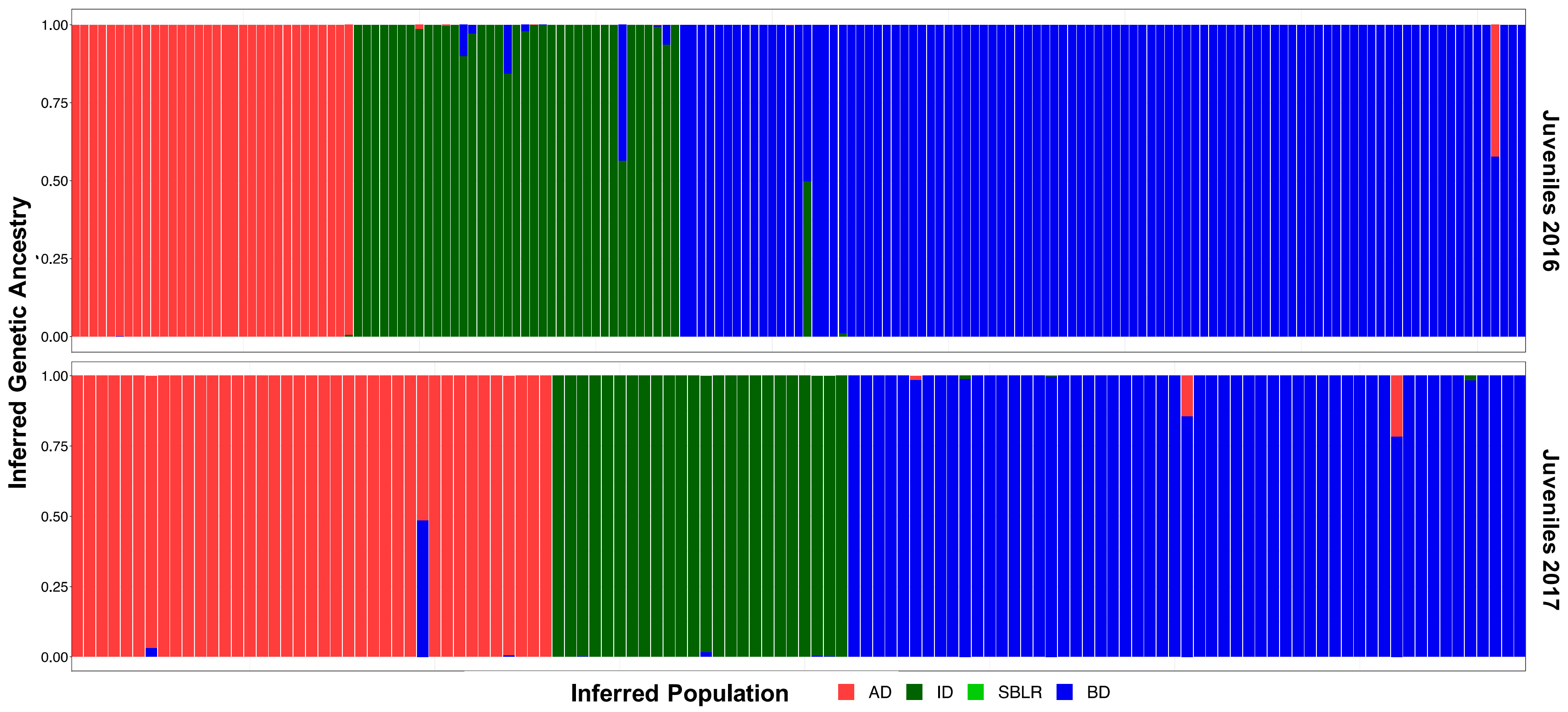

Supplementary Figure 8: The majority of GSI assignments computed by RUBIAS for juvenile smolts collected post dam removal were to BD ID or AD reference collections. No individuals were assigned back to the SBLR. Each vertical bar represents a single individual from one adult cohort labelled along the right y-axis. Colors represented the reference reporting unit or sampling location on the Elwha River

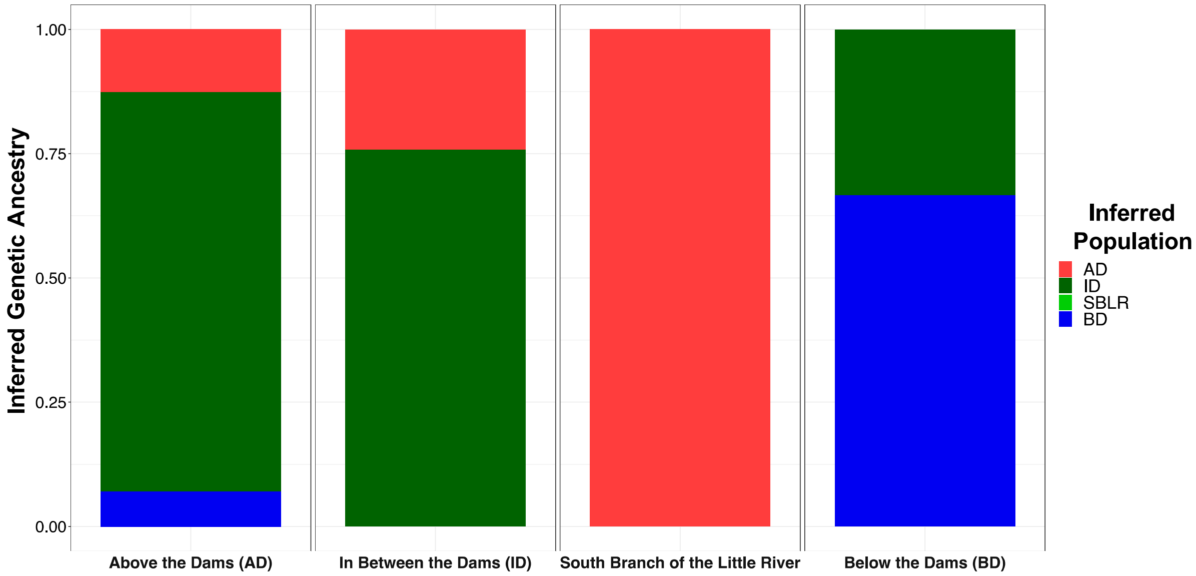

Supplementary Figure 9: Inferred proportions of genetic ancestry from each of the four reference reporting units assigned to the 85 individuals sampled prior to dam removal with discordant DAPC population and sampling location assignments. These samples were assigned to the "prior to dam removal" mixture collection in our GSI analysis of sample set one. Colors are representative of the inferred reporting unit. The bottom x-axis divides sampling locations by relative anadromous barrier location.
